# Caught in the Middle: A Rigid DNA Label That Provides an Incisive Picture of DNA Conformational Flexibility in Protein–DNA Complexes

**DOI:** 10.1021/jacs.5c01823

**Published:** 2025-05-23

**Authors:** Joshua Casto, Shramana Palit, Anthony Little, Zikri Hasanbasri, Linda Jen-Jacobson, Sunil Saxena

**Affiliations:** a Department of Chemistry, 6614 University of Pittsburgh, Pittsburgh, Pennsylvania 15260, United States; b Department of Biological Sciences, University of Pittsburgh, Pittsburgh, Pennsylvania 15260, United States

## Abstract

Measurement of the conformation of DNA in protein–DNA complexes is important to decipher the role of the DNA conformation and dynamics in protein recognition and function. In this work, we report a rigid nucleotide-independent spin label that places paramagnetic Cu­(II) within the DNA helix. The labeling strategy exploits the chelation of Cu­(II) to two 8-aminoquinoline moieties, one in each strand. Because the rigidity of the probe avoids potentially confounding motions of the label itself, EPR signals can resolve 2–3 Å changes in interspin distance; the breadth of the distance distribution reports dynamic fluctuations from the most probable conformation. Continuous wave and pulsed electron paramagnetic resonance spectroscopy (EPR) shows that Cu­(II) coordinates properly to the labeling sites. Measurements of the interspin dipolar interaction on DNA oligonucleotides with two labels placed at various distances demonstrate that the label provides accurate and narrow distance distributions sensitive to DNA conformation and flexibility. Molecular dynamics simulations support these interpretations. We utilized this label to measure the conformations of DNA when the type II restriction endonuclease *Eco*RV binds to its specific recognition sequence. The results provide in-solution evidence that the *Eco*RV endonuclease induces axial DNA bending in the absence of metal ions, contrary to a long-standing belief. Furthermore, the distance distribution narrows upon protein binding and even further on subsequent metal binding, implying that bound protein constrains the bending dynamics of DNA. This method provides a novel and accurate approach to assess DNA conformation and dynamics in solution.

## Graphical abstract

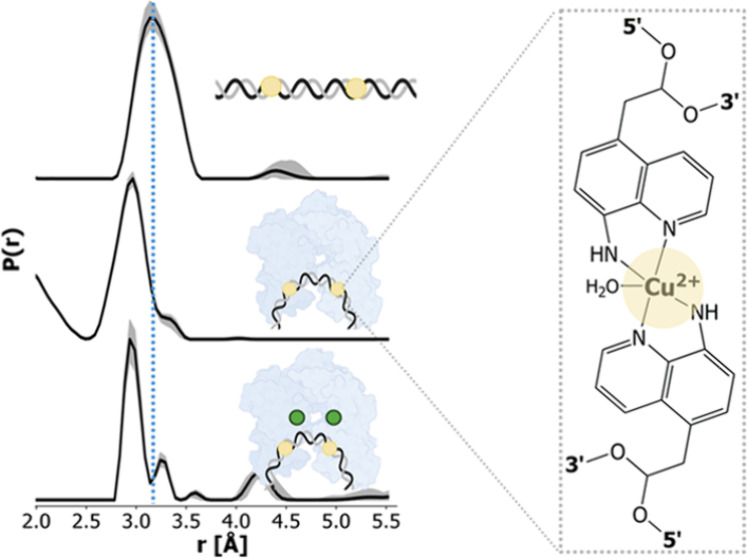

## Introduction

Many physiological processes necessary for survival are mediated by protein and DNA interactions in which one or more proteins bind to a specific sequence in DNA to promote or prevent gene expression, or to modify or cleave DNA. The origin of sequence specific recognition lies in the conformation, flexibility, and dynamics of the DNA.^1–3^ Often, when the DNA binds to a protein, the duplex undergoes some variation of twisting, bending, or a change in flexibility to activate processes such as gene expression or DNA cleavage. Each DNA-binding protein requires some particular set of changes in these properties to accomplish its biological function. For example, changes in DNA conformation and/or flexibility may be required to produce or enhance protein–DNA complementarity, to provide favorable geometry for productive interactions among nearby DNA binding proteins (e.g., in assembling multiprotein complexes to initiate transcription),^4^ or to lower energetic barriers to further dynamic adjustments. “Flexibility” is a collection of properties that characterize the propensity of DNA to alter its conformation. Since such conformational alterations can take many forms, no single parameter of “flexibility” can refer to all of them. Where protein binding causes a departure from the structure and/or dynamics of the protein-free DNA, this makes a thermodynamically unfavorable contribution to protein–DNA interaction, which must be balanced by compensating favorable factors to an extent that the stability of the complex is appropriate to biological function. Thus, capturing these structural transitions of DNA can provide fundamental insight into how physiological processes function.

Experimental approaches to studying these properties have been dominated by methods that yield static structural information, most notably X-ray crystallography. Computational studies (MD simulations) have been very useful as supplements to experiments, although it has proven to be nontrivial to benchmark the accuracy of simulations against experimental data. In principle, spectroscopic methods (e.g., NMR and EPR) offer the potential of providing information about dynamic aspects of the protein–DNA interaction as well as conformational information unobscured by crystal lattice forces.

Electron paramagnetic resonance spectroscopy (EPR) has emerged as an important technique to capture the flexibility and conformational states of DNA and RNA in solution.^5–8^ In these experiments, two spin labels are introduced site-specifically to the nucleic acid via site-directed spin labeling.^9–12^ In general, the labeling site is targeted by attaching a functional moiety to the helix backbone^13–24^ or the terminal ends of the helix^22,25–28^ during DNA synthesis. A spin label is then covalently attached to these functional moieties via a linker. Labeling schemes that incorporate the spin label into the duplex sequence during synthesis have also been reported.^29,30^ However, the use of flexible linkers leads to some limitations.^13,14,17–22,26,29^ The distance distributions measured by EPR are dominated by fluctuations of the linker rather than the duplex backbone. Interpretation of the interspin distance is thus obscured by uncertainties in the (probabilistic) position of the labels.

To address these limitations, rigid nitroxide labels have been developed. For example, the cytidine analogue ç ^31–33^ has been reported on the conformational flexibility and orientations of DNA.^34,35^ Efforts to emulate the power of ç while ameliorating the difficulties of synthesizing the label have also led to the advent of the rigid noncovalent guanine analogue Ǵ ^36,37^ and the isoindoline derivative U labels.^38^ These labels place the spin center outside the helix up to about 1 nm away from the backbone. Thus, modeling is needed to isolate the duplex constraint from the measured distributions. These labels are also nucleotide-dependent and are used to replace the native C, G, or U site in the DNA sequence. Alternatively, Cu­(II) as an EPR probe has been incorporated into guanine quadruplexes via pyridine chelators inside the helix to form a rigid spin label.^39–41^ This scheme is dependent on a quartet of oligonucleotides and requires the incorporation of four Cu­(II) chelators for each labeled site.

Recently, we have developed a sensitive and accurate spin label that binds Cu­(II) specifically to a 2,2′dipicolylamine (DPA) ligand inside the helix.^42–44^ The complementary base pair to DPA is an abasic nucleotide. Notably, this Cu­(II) labeling scheme is nucleotide-independent and is situated inside the helix via a short linker to the backbone.^44,45^ The placement of a spin label close to protein binding sites is advantageous to report on duplex conformations and dynamics in the proximity of the active site of a protein–DNA complex. However, the linker contains two flexible bonds; conformational fluctuations around these bonds result in broad asymmetric distance distributions, and the distance distribution is dominated by the conformation of the linker.^43^ Therefore, there is an ongoing need to develop new spin label technology that circumvents these limitations and enables the precise measurement of DNA shape adaptation and flexibility when binding to protein.

In this work, we have designed a new nucleotide-independent Cu­(II) label for nucleic acids that accurately reports on distance constraints and has narrow distribution widths. For this design, we employed 8-aminoquinoline (AQ). Each AQ has two nitrogen atoms that provide chelation sites for Cu­(II). The labeling scheme is shown in Figure 1. In this approach, two AQ moieties are integrated into the duplex on opposite strands at the desired site to form a complementary AQ pair, which we refer to as AQ_2_. The AQ_2_ site provides four nitrogen atoms for specific Cu­(II) coordination. Each AQ is attached to a phosphoramidite that is incorporated into the helix during DNA synthesis. When Cu­(II) is added to the labeled duplex, it specifically coordinates between the AQ moieties to form a rigidly held spin label in the center of helix–Cu­(AQ)_2_.

**Figure 1:**
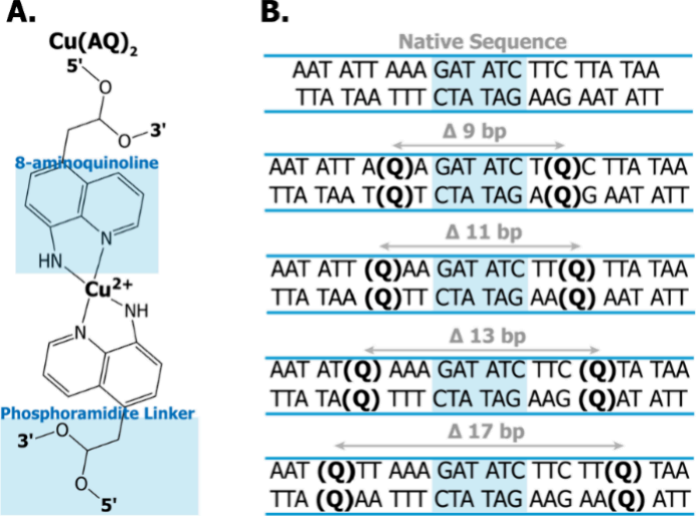
(A) Chemical structure of Cu­(II) coordinated between two 8-aminoquinoline modifications on opposing strands in DNA that compose the Cu­(AQ)­2 motif. (B) The sequences of the unmodified parent duplex and four duplexes that have been modified with 8-aminoquinoline at two sites. The base pair separations range from 9 to 17. The shaded blue region indicates the binding sequence for protein EcoRV.

To demonstrate the utility of the new label, we incorporated it into DNA duplexes that include the GATATC recognition sequence for the *Eco*RV type II restriction endonuclease. The conformational propensities of the *Eco*RV recognition site are dominated by the central T-A step,^46^ as is the recognition site for the TATA box binding protein (TBP) that plays a crucial role in transcriptional initiation in eukaryotes. TBP distorts the TATA box binding site in the form of strong bending into a three-dimensional writhe;^4^ the relatively planar bending of DNA by the *Eco*RV endonuclease provides a simplified exemplar of this.

*Eco*RV is an appropriate system to apply the methodology because of the vast amount of structural and functional information available on the protein–DNA complex.^47–56^ In the presence of catalytic cofactor Mg­(II), the enzyme cleaves DNA in both strands at the center of the GATATC recognition site. Contrary to earlier claims,^57–59^ metal ions are not required for site-specific recognition.^53,55^ However, in the presence of divalent [e.g., Ca­(II)] or trivalent [e.g., Lu­(III)] metal ions that do not support catalysis, *Eco*RV binds to the recognition sequence with higher affinity and selectivity.^53,55,58^ Quantitative binding and footprinting studies showed that *Eco*RV formed a specific complex with its recognition site in the absence of metal ions.^55^ NMR spectra of amide backbone and methyl bearing side chains conclusively showed that most peak positions are distinct for nonspecific and cognate complexes in the absence of metal ion.^53^ Furthermore, chemical shift perturbations in methyl-bearing side chains were observed upon the addition of active site inhibitor Lu­(III) to cognate complexes but not to nonspecific complexes,^53^ suggesting that the metal binding site consisting of both anionic side chains and DNA phosphate is not assembled in the nonspecific complex.

In this article, we first describe the labeling strategy and characterize the Cu­(II) binding by continuous wave (CW)-EPR and pulsed EPR methodology. We then demonstrate the ability of the label to report accurately on DNA conformations and flexibility. We show that the new spin label detects a pronounced conformational change (bending) of the DNA upon binding of *Eco*RV in solution, as previously implied by crystal structures, fluorescence studies, and computational simulations.^46,51,54,56,60^ This bending occurs in both the absence and presence of metal ions.

In our experiments, the *Eco*RV–DNA recognition complex is prevented from entering the catalytic state by the substitution of the catalytic cofactor Mg­(II) with Lu­(III), which mimics Mg­(II) by binding two ions in each catalytic center and acts as a competitive inhibitor (cf. SI Figure S1) but does not support catalysis.

DNA bending (axial flexibility) is essential to the DNA-cleaving activity of *Eco*RV, required to bring the phosphate backbone into apposition with anionic side chains E45, D74, and E77 that together form the binding sites for the divalent metals to support catalysis. These catalytic centers (one in each *Eco*RV subunit) cannot form without the distortion of the DNA imposed by the protein; they are not assembled when *Eco*RV binds to nonspecific DNA. Comparison of experimental DEER distances with distances from *Eco*RV–DNA cocrystal structures shows that the extent of bending of the Cu­(AQ)_2_-DNA is indistinguishable. It has been shown that DNA bending by *Eco*RV is driven by asymmetric neutralization of phosphate negative charges on one face of the DNA by positive charges on *Eco*RV.^56^ Thus, our findings also establish that these electrostatic interactions must be formed correctly when *Eco*RV binds to the spin-labeled DNA.

The breadth of the distribution of distances between Cu­(II) ions provides a measure of the conformational flexibility of DNA. Importantly, we find that this distribution narrows significantly upon *Eco*RV binding, thus providing the first experimental evidence that the DNA dynamics is constrained in the protein–DNA complex. Further narrowing in the presence of the competitive inhibitor Lu­(III) suggests that the enzyme imposed an additional constraint en route to the catalytic transition state.

## Experimental Section

### Cu­(II) Labeling of DNA

We designed complementary oligonucleotides with the basis sequence 5′-AAT ATT AAA GAT ATC TTC TTA TAA-3′ and 3′-TTA TAA TTT CTA TAG AAG AAT ATT-3′. These duplexes contain the specific GATATC sequence that binds *Eco*RV. These constructs were designed to avoid self-complementary hairpin formation. Additionally, the flanking triplet AAA exists in many crystal structures.^50,51,53^ An unmodified base pair was replaced by complementary 8-aminoquinline (AQ) moieties that served to bind Cu­(II). Each modified duplex contained two Cu­(II) binding sites. The positions of substitutions are described in the main text. Modified DNA strands (which had been synthesized, HPLC purified, and characterized by mass spectrometry) were obtained from KareBay Biochem. Duplex DNAs were formed by mixing equimolar amounts of complementary oligonucleotides and annealing in the presence of 2.25 equiv of CuCl_2_ in D_2_O to ensure that all AQ_2_ sites were loaded with Cu­(II). For annealing, samples were heated to 90 °C for 1 min, 60 °C for 3 min, 50 °C for 5 min, 40 °C for 10 min, and 30 °C for 5 min and then cooled to 4 °C using a GeneAMP PCR System 9700. The final Cu­(II) labeled DNA samples were then prepared to 300 μM duplex and 675 μM Cu­(II) in a buffer (pH 7.4) containing 50 mM *N*-ethylmorpholine (NEM) in D_2_O and 40% (v/v) d_8_ glycerol. Deuterated glycerol and water were used to maximize the sensitivity of the pulsed EPR experiments. Free Cu­(II) does not contribute to the signal at pH 7.4 in the NEM buffer.^61,62^ The samples were then placed in 3 mm I.D. and 4 mm O.D. quartz tubes and immediately flash frozen in liquid methylacetylene-propadiene propane (MAPP) gas for X-band measurements.^63^

### Cu­(II)-DPA Labeling of DNA

The single-strand oligonucleotides containing 2,2′-dipicolylamine (DPA) were obtained from ATDBio Ltd. Equal amounts of complementary DNA strands were mixed, and CuCl_2_ was added, ensuring that the Cu­(II) concentration was slightly less than stoichiometric, at approximately 0.94 equiv. of Cu­(II) per DPA binding site. The duplexes were annealed in the presence of CuCl_2_ to promote correct formation and efficient Cu­(II) chelation following the protocol on a GeneAmp PCR System 9700: 90 °C for 1 min, 60 °C for 3 min, 50 °C for 4 min, 40 °C for 4 min, 30 °C for 5 min, and then gradually cooled to 4 °C.

### Preparation of *Eco*RV

Wild-type type II restriction enzyme *Eco*RV was expressed using a pET22b­(+) vector containing the sequence gene in E. coli BL21­(DE3) cells as previously described.^53^ The cell pellets were resuspended in 20 mM Tris-HCl (pH 8.5) with 5 mM NaCl and 0.12% Triton X-100. The cells were lysed by sonification. The lysate was then centrifuged at 4 °C for 30 min at 5000 rpm. The supernatant containing *Eco*RV was first purified with a HiTrap Q HP anion exchange column. The collected flow through was then passed through a GFC size-exclusion column. Purified protein was concentrated in pH 6.5 50 mM sodium phosphate buffer with 150 mM NaCl and stored at −80 °C. Prior to making the final EPR samples, the protein stock was thawed and exchanged into buffer containing 50 mM NEM and 100 mM NaCl in D_2_O (pH 7.4). Samples for EPR experiments were prepared to 175 μM DNA, 400 μM Cu­(II), and 200 μM of *Eco*RV dimer in pH 7.4 50 mM NEM with 40% d_8_ glycerol (v/v) in D_2_O. *Eco*RV was added in slight excess to ensure sufficient binding of all DNA to the protein. For samples containing the *Eco*RV–DNA complex in the presence of metal ion, either 2 equiv. of CaCl_2_ or 4 equiv. of LuCl_3_ per EcoRV dimer was added. Samples were placed in 3 mm I.D. and 4 mm O.D. quartz tubes for X-band measurements and immediately flash frozen in liquid MAPP gas.^63^

### EPR Experiments

Continuous wave EPR (CW-EPR) experiments were performed on each of the duplexes to assess the coordination of Cu­(II) to the duplexes with the AQ_2_ modifications. All EPR experiments in this work were acquired with a Bruker E580 X-Band (∼9.4 GHz) FT/CW spectrometers and Bruker B8692690 cryogen free cryostat. The CW-EPR experiments were conducted using a Bruker ER4118X-MD5 resonator at 80 K. Each spectrum was obtained using a 4 G modulation amplitude, 100 kHZ modulation frequency, 10.24 ns time constant, and 20.48 ms conversion time at an attenuation of 30 dB. Each spectrum was centered at 3100 G and swept by 2000 G over 1024 data points. The collected spectra were then background subtracted, normalized with respect to area, and then simulated using EasySpin.^64^

Electron-spin echo enveloped modulation (ESEEM) experiments^65^ were performed at X-band frequencies using a Bruker EN4118X-MD4 at 18 K. A three-pulse sequence $\left[\left(\frac{\pi }{2}\right)-\tau -\left(\frac{\pi }{2}\right)-\text{T}-\left(\frac{\pi }{2}\right)-\mathrm{echo}\right]$ with four-step phase cycling, a π/2 pulse length of 8 ns, a τ of 136, and an initial *T* of 272 ns was used. The value of *T* was incremented by a step size of 16 ns. Each experiment was performed at the magnetic field of greatest intensity in the field-swept echo-detected spectrum of each Cu­(II) sample. The collected ESEEM trace was phased, background corrected, zero filled with an additional 1024 points, and then fast Fourier transformed to obtain the frequency spectrum. The spectra were then normalized to 1 with respect to the most intense peak.

Pulsed EPR distance measurements were obtained at the X-band using an EN4118X-MD4 resonator and 1 kW TWT amplifier at 18 K. The double electron–electron resonance (DEER) sequence, $\left[{\left(\frac{\pi }{2}\right)}_{\nu 1}-\tau_{1}-(\pi {)}_{\nu 1}-(\tau_{1}+T)-(\pi {)}_{\nu 2}-(\tau_{2}-T)-\pi_{\nu 1}-\tau_{2}-\mathrm{echo}\right]$ with a two-step phase cycling,^66^ and a 10-step tau suppression with an 18 ns step size were used. All samples were optimized to an attenuation and observer frequency that maximized the echo with observer pulses, ${\left(\frac{\pi }{2}\right)}_{\nu 1}$ and (π)_ν1_, of 12 and 24 ns, respectively. The pump pulse, (π)_ν2_, was set to 20 ns and was applied at a −100 MHz offset from the observer frequency. The pulse offset was chosen to minimize the effects of ESEEM on the DEER time trace. For dipolar evolution times (τ_2_) under 2.0 μs, a step size of 12 ns was used. For collections with a longer τ_2_, a step size of 24 ns was used. The pump pulse was applied at the magnetic field of greatest intensity in the echo detected field swept spectrum of each Cu­(II) sample. All measurements were obtained with 100 shots per data point, a 1500 μs shot repetition time, and a Video AMPlifier maximum video bandwidth set to 20 MHz. The echo integrator gate length was set as the full width at half-maximum of the DEER echo. Integrator gate lengths varied between ∼30 and 40 ns depending on the operating frequency and magnetic field position of the respective measurement. The DEER time traces were then analyzed using DEERAnalysis^67^ to determine the distance distributions and standard deviations of the most probable distribution. The background of the primary DEER data was corrected in DEERAnalysis using a second-order polynomial as is appropriate for Cu­(II)-DEER.^68^

### Molecular Dynamics Simulations

The B-DNA model using the unmodified parent sequence of the labeled duplexes was created using the Nucleic Acid Builder program in the AMBER software suite.^10^ We performed five replicates of 1 μs production simulations. The DNA was solvated in water using the TIP3P water model in a 14 Å truncated octahedral water box and neutralized with 46 Na^+^ ions.^69^ Molecular dynamics (MD) simulations were performed on the model using the AMBER parmbsc force field in the AMBER20 software package.^70^ To energy minimize the solvated model, the system was thermalized from 0 to 298 K with a constant restraint force on the entire NDA molecule at 1.0 kcal mol^–1^ Å^–1^ over 20 ps. The system was equilibrated at 298 K at a constant pressure of 1 atm for 1 ns with constant strain and then 1 ns without restraint. The integration time step for heating, equilibration, and production runs was 2 fs. During production runs, periodic boundary conditions with a particle mesh Ewald^71^ were applied to account for long-range electrostatic interactions under NPT (P-1 atm) conditions. SHAKE^72^ was used to restrain all bonds involving hydrogen using a nonbond cutoff of 10 Å. The distance measurements of the MD trajectories from the five replicates were obtained by using CppTraj and then averaged together.

## Results and Discussion

### Label Coordination and Geometry

Figure 1A shows the chemical structure of the label with Cu­(II) coordinated equatorially to four nitrogen atoms from two 8-aminoquinoline (AQ) moieties on opposite strands of the DNA. The volume of this new Cu­(AQ)_2_ label is ca. 278.2 Å^3^ compared to 268.7 Å^3^ for AT and 257.0 Å^3^ for GC nucleotide base pairs.^73^ Figure 2B shows the sequences and base pair separations between the labeled sites for each of the designed duplexes and the unmodified parent duplex. The highlighted blue region is the specific EcoRV recognition sequence.

**Figure 2:**
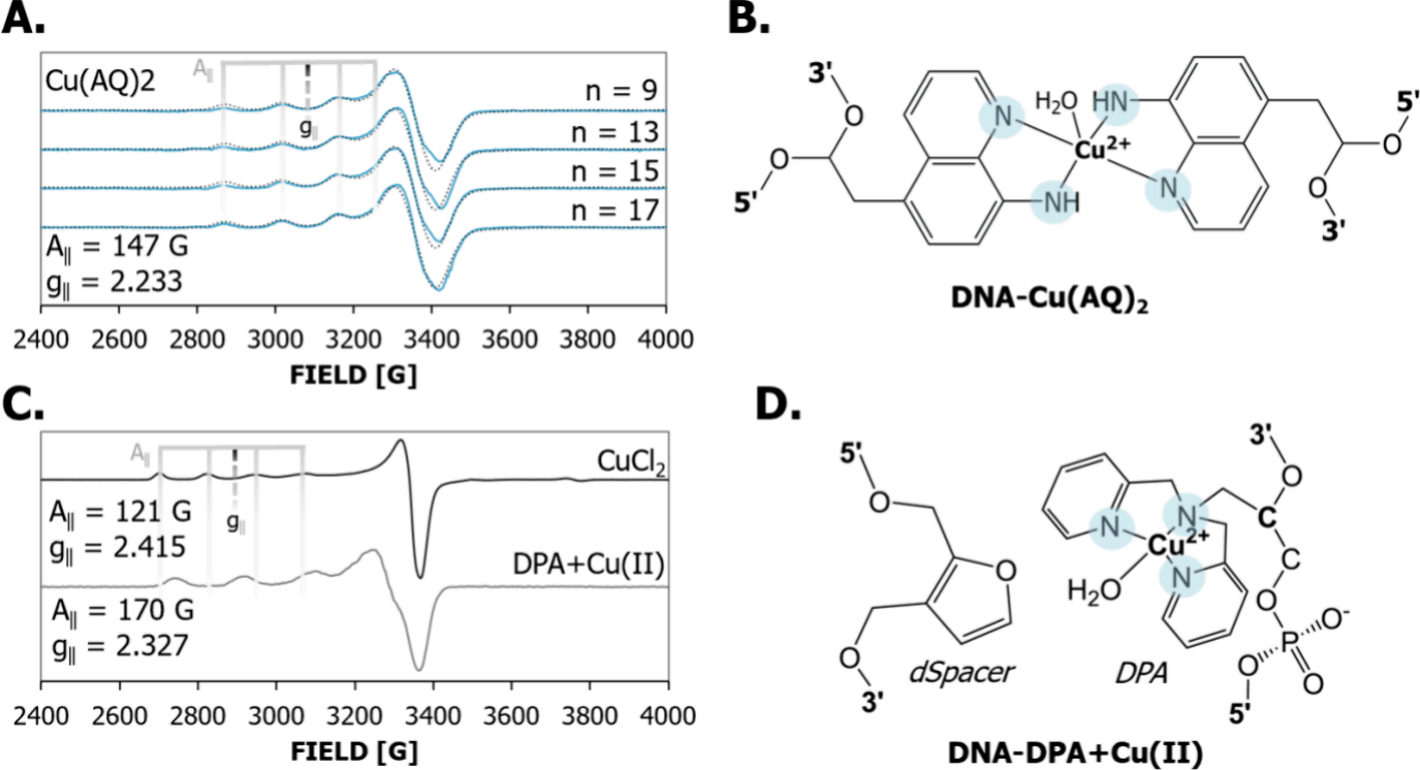
(A) CW-EPR spectra for the four Cu­(AQ)_2_ duplexes are shown. All duplexes (blue) have one component spectra with an *A*_∥_ of 147 G and *g*_∥_ of 2.233. The simulated spectra are shown by dotted lines. Gray bars and the black dashed line indicate *A*_∥_ and *g*_∥_, respectively, of Cu­(AQ)_2_ to facilitate comparison to the other spectra. (B) Chemical structure of the Cu­(AQ)_2_ label. The four coordinated nitrogen atoms are highlighted. (C) The CW-EPR spectra of CuCl_2_ and DNA labeled with DPA. (D) Chemical structure of the DPA label with the complementary dSpacer. For CuCl_2_ (black), *A*_∥_ is 121 G and *g*_∥_ is 2.415. Gray bars and the black dashed line indicate *A*_∥_ and *g*_∥_, respectively, of CuCl_2_ for comparison to the other spectra. The DPA label (gray) has an *A*_∥_ of 170 G and *g*_∥_ of 2.327. The lower *g*_∥_ in the AQ_2_ duplexes is consistent with an increase in directly coordinated nitrogen atoms upon loading to AQ_2_ compared to free Cu­(II).

### CW-EPR Suggests That Cu­(II) Coordinates to Target Sites in the DNA

We first performed CW-EPR to show that Cu­(II) specifically coordinates to the targeted sites in each duplex. The spectra were obtained in the NEM buffer where Cu­(II) is EPR silent.^72^ Figure 2 shows the 80 K CW-EPR data obtained on the labeled duplexes, CuCl_2_,^74–79^ and a duplex labeled with the previously developed DPA label.^42–44^ The four “splittings” due to *A*_∥_ are indicated by gray lines on the CW-EPR spectra in the 2800–3200 G range. Notably, all Cu­(AQ)_2_ labeled duplexes have similar one-component spectra. The simulated spectra are also overlaid on the experimental data in Figure 2A. The measured values of *g*_∥_ and *A*_∥_ of 2.232 and 147 G, respectively, are in the range of complexes with Cu­(II) coordinated equatorially to four nitrogen atoms.^78^

Figure 2C shows the spectra for the CuCl_2_ and DPA DNA Cu­(II) label for comparison. The peak positions for CuCl_2_ and the DPA DNA label are different from those observed for the newly developed Cu­(II) label. The changes in the values of *g*_∥_ for Cu­(II)­AQ_2_ compared to those of CuCl_2_ and Cu­(II)-DPA are consistent with an increase in the number of directly coordinated nitrogen atoms,^74,80^ which alters the spin orbit coupling.^74^

Notably, the *A*_∥_ of Cu­(II)­AQ_2_ labeled DNA is markedly lower than the *A*_∥_ value of 170 G observed for the DPA Cu­(II) label. The decrease in *A*_∥_ is surprising given the higher number of coordinated nitrogen atoms for AQ_2_ compared to DPA. Indeed, the *A*_∥_ and *g*_∥_ are outside the typical range of square planar and octahedral complexes with four equatorially coordinated nitrogen atoms.^74^ Therefore, the coordination geometry for Cu­(II)­AQ_2_ in DNA is likely not square planar. Square pyramidal and distorted tetrahedral Cu­(II) complexes coordinated with equatorial nitrogen atoms have been reported with lower *A*_∥_ values than octahedral and square planar counterparts.^81,82^ Thus, a water or other molecule may also be coordinated axially to copper (discussed in the next section). The aromaticity and overall net charge of the Cu­(II)­AQ_2_ may also play a role in the reduced hyperfine value.^74^ Taken together, the CW-EPR data strongly suggest that the Cu­(II) is specifically coordinated to both 8-aminoquinoline moieties in the duplex.

### Pulsed EPR Confirms That Cu­(II) Binds to Target Moieties

To obtain further insights into coordination, we performed electron spin echo envelope modulation (ESEEM) experiments.^65^ ESEEM is sensitive to nuclear quadrupole interactions and hyperfine interactions between Cu­(II) and remote nuclei within approximately 3–8 Å.^65^ Figure 3A shows the ESEEM signal for two of the duplexes, and the spectra are shown in Figure 3B. Both time traces have identical modulations. For both duplexes, we observed peaks at ca. 2.3 MHz and at ca. 14 MHz. The peak at ca. 14 MHz is characteristic of proton interactions. The peak at ca. 2.3 MHz with broad shoulders is characteristic of deuterium. This peak contains two main features: a doublet centered around 2.3 MHz with a full width at half-maximum of ca. 0.5 MHz and a broad shoulder that goes from 0.2 to 4 MHz. The doublet at 2.3 MHz arises from the weak nuclear quadrupole interaction of deuterium in D_2_O with Cu­(II).^83^ Additionally, the broad shoulder from 0.2 to 4 MHz is characteristic of deuterium from the axially bound D_2_O to Cu­(II).^83^ The small peak at 4.5 MHz is characteristic of the sum combination Larmor frequencies of the deuterium transitions.^84–87^ Notably, the ESEEM does not show any peak from nitrogen, which would lead to three lines below 2 MHz and a broad peak at around 4 MHz.^88–93^ Additionally, ESEEM in the absence of D_2_O was performed (cf. Figure S2). The data did not show peaks corresponding to the weakly coupled nitrogen from adjacent base pairs. These results provide strong evidence that all four nitrogen atoms from the two AQ moieties directly coordinate to Cu­(II) in the duplex. If one of the nitrogen atoms was not coordinated to Cu­(II), then nitrogen ESEEM would be present.

**Figure 3:**
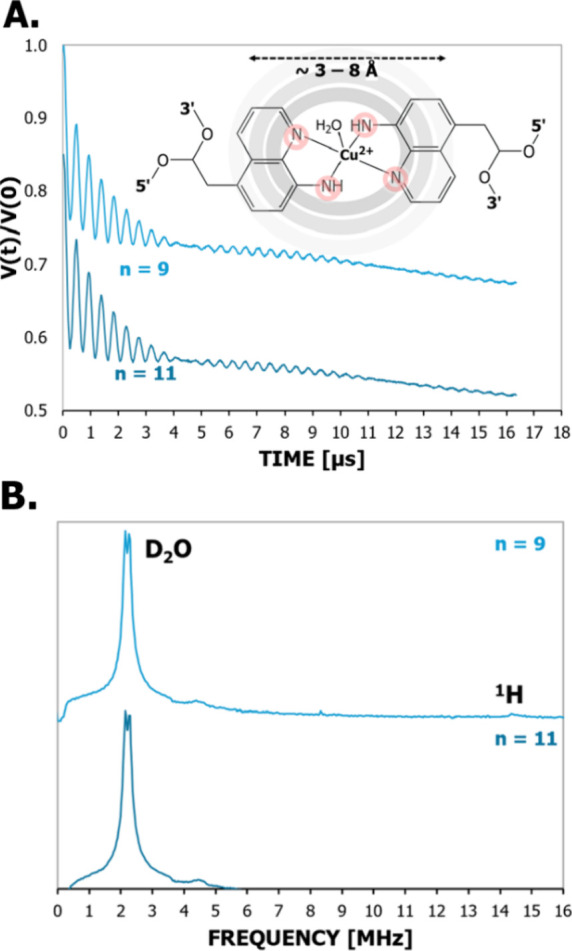
(A) The ESEEM signal for the CuAQ_2_ modified duplexes with 9 and 11 base pair separations. ESEEM is sensitive to nuclear spins between 3 and 8 Å of the spin center. Both duplexes have identical modulating ESEEM signals that are characteristic of deuterium. There are no oscillations in the signal indicative of nitrogen. (B) The fast Fourier transformed ESEEM spectra of the two duplexes. There are peaks characteristic of deuterium and protons present at ca. 2.3 and 14 MHz. The deuterium peak is composed of a narrow doublet at ca. 2.4 MHz and a broad shoulder that spans from 0.2 to 4 MHz. The broad shoulder is due to hydrogen bonding from a D_2_O coordinated to Cu­(II), while the narrow peak with a doublet ca. 2.3 MHz is due to remote D_2_O. The weak nuclear quadrupole interaction of deuterium in D_2_O causes the doublet. The narrow breadth of the 2.3 MHz doublet is affected by the formation of a D_2_O complex with the spin center. The full width at half-maximum breadth of ca. 0.5 MHz for the 2.3 MHz doublet is indicative of axial D_2_O coordination. The lack of the three sharp nitrogen peaks below 2 MHz and a broad peak at 4 MHz indicates that there is no uncoordinated nitrogen near the Cu­(II) center. Taken together, the ESEEM data support that Cu­(II) is coordinating with the four nitrogen atoms from AQ_2_ equatorially and D_2_O in at least one axial position.

The ESEEM data further confirm that Cu­(II) properly coordinates to the four target nitrogen atoms of the chelating 8-aminoquinoline moieties in the duplex. In addition, it is likely that there is at least one axial D_2_O. Cumulatively, these results combined with CW-EPR data (cf. Figure 2) suggest that the label has a square pyramidal or distorted tetrahedral geometry.

### Distance Measurements Show That the Label Is a Sensitive and Accurate Probe of DNA Conformation and Flexibility

Next, we performed X-band DEER on each of the duplexes. In this work, the data were acquired at X-band frequencies (∼9.5 GHz) even though operating at Q-band frequencies (∼32.4 GHz) significantly increases signal sensitivity for distance measurements.^94–96^ This choice was made because rigid copper labels can exhibit orientation selectivity in Q-band measurements.^97–99^ While such effects have been observed even at the X-band for constrained systems,^100,101^ previous work on peptide nucleic acids with similarly rigid Cu­(II) label did not display orientation selection.^102^ Thus, measurements at the X-band potentially avoid complications in the interpretation of distance distributions.^103,104^

Figure 4A shows the background-corrected time traces and the fits obtained with DEERAnalysis.^67^ Each time trace has an SNR greater than 20 per published EPR spectroscopy community guidelines for reporting and interpreting distance distributions.^105^ The primary data for the duplexes are provided in Figure S3 in the SI. The pump pulse in these measurements was applied at the maximum of the echo detected field swept spectrum of Cu­(II). Additional DEER measurements across the FS-ESE spectrum were performed for the *n* = 9 DNA to confirm that the data are not orientation selective at the X-band (Figure S4). Comparison of the Pake patterns with simulations (cf. Figure S5) also indicates proper orientational sampling. These results are not surprising in light of earlier X-band work on similarly rigid labels.^102,104,106^ In such labels, the Cu­(II) coordination is elastic, and there exist a range of bond lengths and bond angles for the bonds that involve the central metal ion. This elastic coordination leads to a range of orientations of the *g*-tensor axis leading to a large distribution in the relative orientations of the two *g*-tensors in a bilabeled molecule.^102,103,107^ Thus, a large range of interspin vectors can be sampled at a given field at X-band even though the metal ions are held rigidly in position.

**Figure 4:**
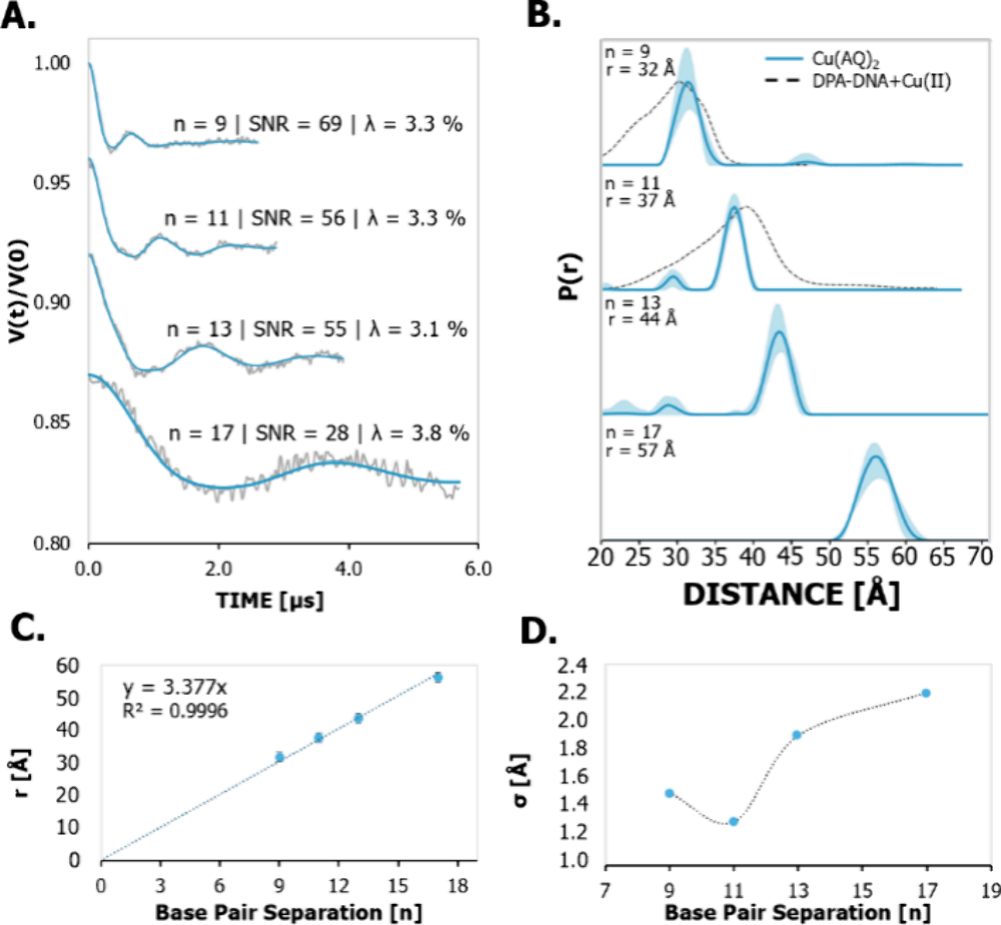
(A) Background-corrected DEER time traces for the duplexes. The SNR was calculated by dividing the modulation depth by the RMSD of the residuals. The blue lines are fits obtained by DEERAnalysis2022. There are clear changes in the modulation period as the base pair separation between the Cu­(II) sites increases. (B) Distance distributions for the duplexes. The distance distributions were obtained with DEERAnalysis via Tikhonov regularization and background validation. The most probable distance increases as the base pair separation between the Cu­(II) labeled sites increases. For comparison, the distance distribution using the DPA label is also shown as a dashed line for the *n* = 9 and *n* = 11 duplexes. The Cu­(AQ)_2_ has much narrower distributions than DPA-Cu­(II). (C) The most probable distances are plotted as a function of *n* with the *y*-intercept set to 0. The slope is 3.4 Å, which agrees with the estimated 3.4 Å between base pairs for B-DNA. (D) The standard deviation of the distance distributions plotted as a function of base pair separation, *n*. These results indicate that the Cu­(II) label reports directly on the DNA conformation.

The data traces in Figure 4A show a clear increase in the modulation period as the base pair separation between the labeled sites increases, which suggests an increase in distance with separation. Moreover, Figure 4B shows the validated distance distributions obtained from DEERAnalysis.^67^ The most probable distance for each DNA is consistent with observed modulation periods (cf. Table S2 in the SI for details). Figure 4C shows the most probable distance plotted as a function of base pair separation. A fit to a straight line yields a *y*-intercept of 0 and a slope of 3.4 Å. This slope is consistent with the expectations based on the rise between base pairs of a B-DNA helix.^108^ Figure 4B also compares the distance distributions of the new Cu­(II) label to those obtained using the DPA label for *n* = 9 and *n* = 11. The new label has a most probable distance within a few angstroms of the DPA Cu­(II) label distance. This result is consistent with the placement of Cu­(II) within the helix in both labeling schemes. Given that the Cu­(II)–Cu­(II) distance of the DPA Cu­(II) label can be directly related to the C4’–C4’ and C3′–C3′ backbone distances,^43^ we anticipate that the most probable distance from the new labeling scheme is similarly an accurate reporter of average DNA conformation.

Notably, Figure 4B shows that the distribution width for the new label is dramatically narrower than the distribution of the DPA label. Here, the standard deviations are used to describe the breadth of the distributions. The distribution breadth for the new label has a standard deviation of 1.5 to 2.2 Å depending on the interspin distance, whereas the distribution for the DPA label has a standard deviation of approximately 4 Å.^44^ In comparison, the rigid nitroxide ç label has a distribution with standard deviations between ca. 1.5 and 4 Å for base pair separations similar to the duplexes presented in this work.^35^ Such narrow distributions indicate that Cu­(II) is reporting primarily on the flexibility and conformations of the duplex rather than being biased by the flexibility of the label linker. The narrow width for the new Cu­(II) label can be attributed to the use of two moieties to “anchor” the Cu­(II) inside the DNA helix to reduce its mobility. The distribution width decreases from base pair separations of *n* = 9 to 11 and then increases again for *n* = 13 and 17. Such variation in the distribution widths is not unusual and has been previously shown to be due to breathing modes of DNA dynamics of such lengths.^35,109,110^ The Supporting Information details a breathing model that analyzes such a trend in the distributions (cf. Figures S7 and S8). The breathing model also shows that the new label is less flexible than the DPA Cu­(II) and ç labels. Details of the analysis and comparison are presented in the SI (cf. Figures S7 and S8). These results suggest that the use of two 8-aminoquinoline residues provides a label that directly measures distance constraints that are accurate reporters of DNA conformation and flexibility.

### MD Simulations Confirm That the Label Is an Accurate Reporter of DNA Conformations

We performed five replicate 1 μs molecular dynamics (MD) simulations to compare the experimental Cu­(II)–Cu­(II) distance distributions to the expected backbone distribution. In these MD simulations, the unmodified parent DNA sequence was used (Figure 5A). We analyzed the MD trajectories (5 μs in total) to extract a distance distribution that corresponds to the base pair separations. This distance is illustrated in Figure 5A for the *n* = 9 duplex. For this duplex, we extracted the distance between the hydrogen atom closest to the center of the helix on the thymine at positions 8 and 17. Similar choices were made for other values of *n*. In instances where a CG base pair was present, the hydrogen on guanine closest to the center of the helix was used for distance measurement.

**Figure 5:**
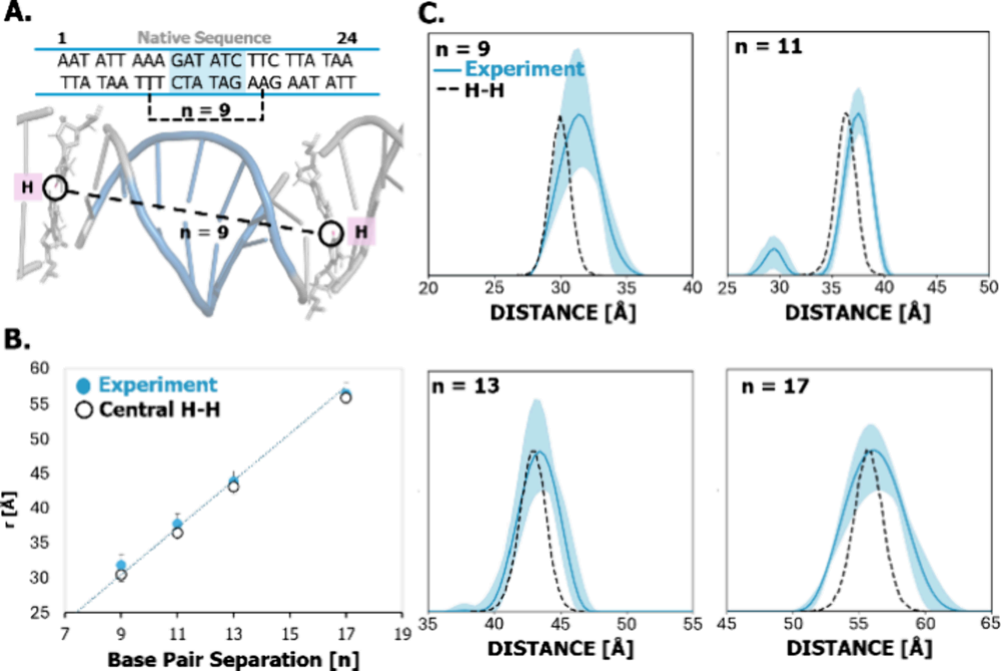
(A) Representative structure of the *n* = 9 DNA sequence from one frame of the five replicate 1 μs MD simulations. For this duplex, the distance between the hydrogen atom on thymine (H3) at each labeled site was used to measure the distance distribution from MD. This distance and the nucleotides are shown in the figure. In the labeling scheme, these nucleotides are replaced by AQ2, and the chosen H atoms are closest to the central point between complementary base pairs and are therefore good surrogates for the Cu­(II)–Cu­(II) distance. Similar choices were made for other values of *n*. (B) Comparison of the most probable experimental distance and H–H measurements from MD plotted as a function of base pair separation *n*. The distances are within ∼1.5 Å. Such agreement is consistent with Cu­(II) being placed in the center of the helix. (C) Comparison of the H–H backbone distance measurements from MD (black dashed line) to the experimental Cu­(II)–Cu­(II) distance distribution from Tikhonov regularization (blue). The distance distribution widths are remarkably similar. These results show that the Cu­(II) label can accurately report on DNA distance restraints.

These terminal H atoms on nucleotides were selected because they are between base pairs that are replaced with AQ_2_ and best approximate the location of Cu­(II) in the labeled duplexes. Figure 5B shows the experimental Cu­(II)–Cu­(II) distance distributions plotted against the H–H MD distances as a function of base pair separation. Notably, the experimental distances are all within 1.5 Å of the H–H distance across base pair separations. These results reinforce how precisely the new Cu­(II) label reports on the B-DNA conformations and affirms the positioning of Cu­(II) in the center of the helix. Figure 5C shows the experimental and H–H distributions for the duplexes. Here we see that the experimental widths are in reasonable agreement with distributions obtained by MD.

### Changes to DNA Conformation and Flexibility in *Eco*RV–DNA Complexes

EPR measurements were then performed on DNA in the presence of *Eco*RV with and without metal ions (cf. Figure 6). The data were acquired for the *n* = 9 duplexes. Each labeled duplex contains the specific *Eco*RV recognition sequence GATATC (cf. Figure 1).

**Figure 6:**
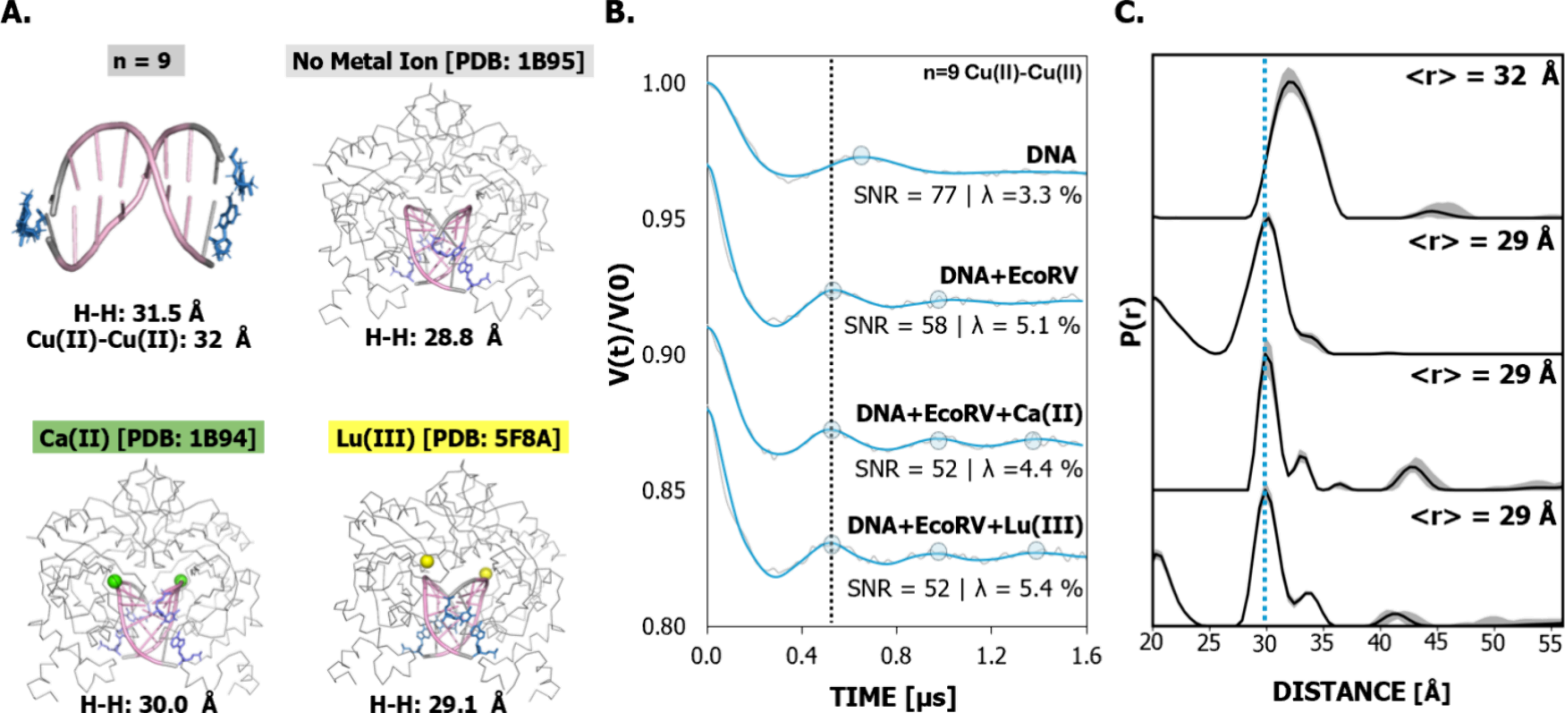
(A) Comparison of the *n* = 9 duplex structure obtained from MD to the crystal structures of DNA bound to *Eco*RV [PDB: 1B95], DNA bound to *Eco*RV coordinated to Ca­(II) [PDB: 1B94], and DNA bound to *Eco*RV coordinated to Lu­(III) [PDB: 5F8A]. These crystal structures were chosen because they use the same binding sequence in DNA as our labeled duplexes. (B) DEER distance measurements for the *n* = 9 duplex in the presence of *Eco*RV, and *Eco*RV coordinated to Ca­(II) or Lu­(III). The background-corrected DEER time traces (gray) and the fits (blue) obtained via Tikhonov regularization in DEERAnalysis. The black dashed line is used to emphasize the shortening of the time trace modulation period as *Eco*RV is added to the labeled duplex. A shortening of the modulation period is indicative of a decreasing distance. (C) The distance distributions for each of the DNA samples as shown. The most probable distance decreases by 3 Å when the DNA is in the presence of *Eco*RV or *Eco*RV coordinated to Ca­(II) or Lu­(III). The gray shaded regions are the validated distance distributions. The distances from the crystal structure were calculated using the measurement tool in PyMol.^109^

In the active state, *Eco*RV coordinates two Mg­(II) in each of the two active sites of the dimeric complex to carry out cleavage of the two strands of DNA.^48,54^ One Mg­(II) is responsible for neutralizing the negative charge of the DNA backbone to improve the ability of *Eco*RV to bend the DNA.^48,111^ The other Mg­(II) activates a water to hydrolyze a phosphate in the duplex to catalyze the cleavage of the DNA.^48,54,111^ Studies of the basis for protein–DNA binding specificity have often relied on the use of Ca­(II) or Lu­(III) as metal ions; both act as competitive inhibitors of Mg­(II)-induced DNA cleavage and improve *Eco*RV–DNA binding (Figure S1). Ca­(II) or Lu­(III) occupies both catalytic centers; however, NMR titration shows that Lu­(III), like Mg­(II), places two ions in each center.^48,53,54,112^ On the other hand, crystal structures show only one Ca­(II) in each catalytic center.^113^ The new EPR probe permits elucidation of how the noncatalytic complexes containing metal ions affect DNA conformation.

Figure 6A shows the MD equilibrated duplex and also the crystal structures of DNA bound to *Eco*RV and of the protein–DNA complex with Ca­(II), and Lu­(III). The positions of the bound metal ions are shown as spheres. The *n* = 9 DNA distances in the crystal structures were obtained between the H atoms of thymine that are closest to the center of the helix. This H–H distance measurement denoted in Figure 6A was chosen to emulate our MD analysis and Cu­(II) positioning in the duplex. In the EPR measurements, the DNA was labeled with Cu­(II) prior to the addition of the protein. Figure 6B shows the background-corrected DEER traces and fits for the *n* = 9 duplex in the presence of *Eco*RV and in the presence of *Eco*RV with Ca­(II) or Lu­(III). The primary DEER data are shown in Figure S3. There is a clear reduction in the modulation period in all protein–DNA complexes compared to free DNA. The dashed line in Figure 6B highlights the clear change in the modulation period between the free duplex and the duplex in the presence of the protein. The extracted distance distributions for these complexes are shown in Figure 6C. The most probable distance decreases by 3 Å in the presence of the protein with and without metal ions. The primary data were also analyzed with ComparativeDEERAnalyzer^114^ (cf. Figure S9) and shows agreement with the DEERAnalysis results in Figure 6C. A shortening of the distance is indicative of *Eco*RV inducing a conformational change in the duplex in the form of a bend. The Cu­(II)–Cu­(II) distances are nearly identical to the H–H distances in the crystal structures, indicating very similar degrees of protein-induced DNA axial bending. Note that DNA is not bent in the crystal structures of nonspecific *Eco*RV–DNA complexes. Thus, the data show that the Cu­(II) label can report on conformational changes in the range of a few angstroms

These data highlight the sensitivity of the new Cu­(II) label; a ca. ∼200 ns change in modulation period corresponding to a 3 Å change in distance is clear in the traces (cf. Figure 6B). Importantly, the change in distances is consistent with the distances obtained from the crystal structures (cf. Figure 6A).^53,113^ This result shows that the label reports *accurately* on the DNA conformation without convolution due to flexing of the probe itself. Moreover, the measured thymine H–H distances are similar in the crystal structures of *Eco*RV–DNA complexes with or without Ca­(II) or Lu (III) (cf. Figure 6A).

Notably, the breadth of the distance distributions is narrower for DNA when bound to *Eco*RV and metal ions relative to that for the free DNA. The addition of *Eco*RV leads to a remarkable decrease in the flexibility of the DNA (Figure 6C). This reduction in the distribution width is easily observable in the time trace (Figure 6B). The peak positions of the oscillations are highlighted by circles in the time traces shown in Figure 6B. Comparing the time traces of free DNA and the *Eco*RV–DNA complex shows that the presence of *Eco*RV causes an additional modulation period to be visible. Additionally, the oscillations become more pronounced, up to three modulation periods, in the presence of the Ca­(II) and Lu­(III) inhibitors.

In addition, there are features around 32–40 Å present in some of the distance distributions. The origin of these features is unclear, but they could be due to difficulties with background suppression for these challenging data sets that display a large modulating signal even at longer acquisition times. Further, these experiments were conducted with a small excess of Cu­(II). ESEEM and CW-EPR results suggest that Cu­(II) can bind to *Eco*RV (cf. Figures S10–S12). The binding of Cu­(II) to *Eco*RV likely results in distances between 32 and 40 Å observed in Figure 6C. It is possible that Cu­(II) binds to an amino acid proximal to the active site as it does for *Eco*RI.^115^ Therefore, distance measurements may capture a small population of Cu­(II)–Cu­(II) distance within the protein and/or between the labeled DNA sites and the protein.

### Insights into the Structure and Dynamics of *Eco*RV–DNA Interactions from EPR

In computational studies, free DNA containing the GATATC recognition site is unbent, and a crystal structure of a nonspecific EcoRV complex shows unbent DNA.^46,47^ However, we observe, in the presence of protein, a reduction of 3 Å for the most probable Cu­(II)–Cu­(II) distance, indicating that *Eco*RV bends the DNA axially to bring the DNA backbone toward the active site on the protein to cleave the duplex.^47,51^ Thus, DEER measurements with this label capture the bent DNA conformation of a specific bound complex in solution. Overall, the comparison of the most probable distance from the *n* = 9 duplex in the presence of *Eco*RV and with distances for these positions in crystal structures indicates that the DNA in *Eco*RV complexes is bent to a similar degree in solution.

It is noteworthy that we observe a bent DNA distribution in the presence of a bound protein alone. It was at first suggested that EcoRV binds to specific and nonspecific DNA sequences with equal affinity in the absence of metal ions^52,59^ and that the coordination of metal ions to the *Eco*RV–DNA complex causes a switch from a nonspecific binding state (with unbent DNA) to a specific binding state.^57,58^ However, Figure 6 shows that the most probable distances in the presence of a protein are the same with or without metal ions. Therefore, the EPR data show that EcoRV bends the DNA with or without metal ions. DNA bending without metal was also suggested previously by NMR, fluorescence studies, and computational simulations.^46,53,56,60,111^

Figure 7 quantifies the widths of the distance distributions of the free duplex and the DNA in protein complexes with and without metal ions. The width of the distance distribution measures, for an ensemble of protein–DNA complexes, the variation of the sampled ensemble conformations from the most probable distance. That is, a broader distribution indicates an increased DNA flexibility in the *Eco*RV–DNA complex. The flexibility that affects the distance distribution perhaps reflects two partly distinct properties: (a) flexing at the central DNA bend, which affects the distance between spin labels outside the recognition site, and (b) conformational fluctuations in regions beyond the central bend, affecting primarily the widths of the distance distributions.

**Figure 7:**
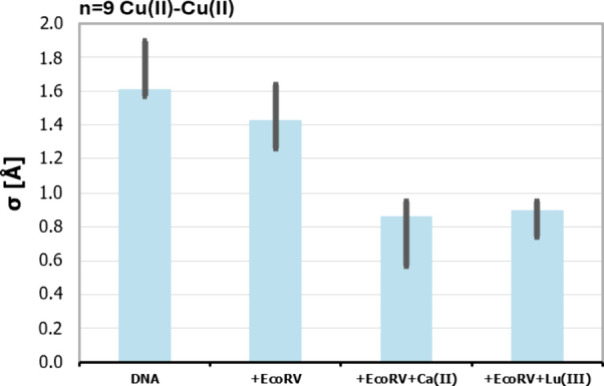
Comparison of the standard deviations in the distance distributions for the (A) *n* = 9 duplexes for unbound DNA and in the presence of *Eco*RV with or without Ca­(II) and Lu­(II). As a rough estimate, the errors (gray) of the standard deviations were calculated by taking the difference between the highest and lowest standard deviation from the validated distance distributions (cf. gray in Figure 6C). For both duplexes, the standard deviations of the Cu­(II)–Cu­(II) distances in DNA decrease in the presence of *Eco*RV, indicating that the duplex rigidifies. The Cu­(II) label can therefore detect subtle changes in DNA flexibility in response to complex formation and biomolecular assembly.

Figure 7 shows a decrease in the distribution width of the *n* = 9 duplex in the presence of EcoRV relative to the free DNA. This indicates that the axial fluctuations in the DNA are constrained by EcoRV. Note that the spin labels are placed in the regions flanking the GATATC binding site, and thus, our results indicate that DNA dynamics beyond the recognition sequence is altered upon protein binding. This constraint on fluctuations in the axial bend has thermodynamic consequences as an unfavorable decrease in internal entropy, presumably compensated by an improvement in enthalpy as a consequence of improved protein–DNA complementarity that optimizes protein–DNA hydrogen bonds, salt links, etc.

Moreover, for the *n* = 9 duplex, the widths of the distance distributions decrease further when Ca­(II) or Lu­(III) is added. This latter observation suggests that metal ion neutralization of the constellation of repulsive negatively charged carboxylate side chains and DNA phosphate at the active site imposes further restriction of DNA dynamics. The response to metal ions shows that the chelating functional groups in the (CuAQ)_2_–DNA–*Eco*RV complex are properly assembled into metal-binding sites,^53^ a property that for the parent unmodified DNA is uniquely associated with sequence-specific recognition^53^ and DNA bending. Note that for the *n* = 9 duplex, the Cu­(II) atoms lie entirely within the DNA region engaged in a direct interface with EcoRV. It is remarkable that the new EPR probe appears to resolve this dynamic boundary in the protein-bound DNA. The trends in the distribution breadth presented in Figure 7 are preserved even when augmenting the regularization parameter used in the DEER time trace fitting analysis (cf. Figure S13). The EPR results show that the changes in DNA flexibility at the central bend in the metal-free *Eco*RV–DNA complex are observable at two base pairs outside the specific protein binding sequence (cf. Figure 1), but the further constraint due to metal ions is not.

It would also be beneficial to assess the changes in the dynamics of the protein of the complex to complement these observations. Supplementary experiments with a nonspecific DNA sequence are also necessary to further assess the relationship between DNA flexibility and specific binding.

It is instructive to compare the AQ_2_ label to existing labeling methodology for DNA. Conceptually, this label places the EPR reporter in the middle of the DNA helix, a property that is shared with DPA. Our results show the enhanced resolution of the AQ_2_ label over DPA. Nitroxide-based labeling, on the other hand, attaches the EPR reporter away from the helix by using a linker of ca. 1 nm length. The AQ_2_ label measures the most probable distances that provide a direct measure of the DNA conformation. Indeed, rigid Ǵ,^36,37^ ç,^31–33^ and semirigid R5c^16^ labels can provide similar results on *unbent* DNA as long as the two labels are colinear on the same face of the DNA helix.^35,36^ On the other hand, the length of the linker needs to be accounted for if the labeling sites are not on the same face.^36,116^ Such analysis is easier for rigid labels, whereas modeling procedures are typically utilized for flexible labels. A comparison of measured distances using the different DNA labels is provided in Table S3 in the SI.

Second, the Cu­(II) label yields narrow distance distributions, a property that it shares with Ǵ and ç, which is useful for the measurement of function-related changes in DNA conformation and flexibility. There is limited literature utilizing rigid Ǵ and ç labels to probe conformational changes in DNA upon protein binding. Noncovalently bonded ç has been used to observe DNA bending in the presence of protein.^117^ In this work, the spin labeling efficiency was reduced to ca. ∼20% due to the close proximity of the labeled site to the protein recognition site. In comparison, we show herein that the copper label observes protein-induced changes in conformation and flexibility, with a probe placed only two base pairs away from the recognition site (cf. Figure 1, *n* = 9). Indeed, the observation of bending indicates that the label does not perturb protein recognition to the target DNA sequence. The EPR label also yields a clear delineation of the protein-induced restriction in the conformational vibrational flexibility of the DNA.

Flexible nitroxide-based DNA labels have been used to investigate protein–DNA interactions.^118,119^ Here, modeling techniques^120^ are required to deconvolute the interpretation of the distance distribution in terms of DNA conformation, and it is more difficult to assay the DNA flexibility. The AQ_2_ label confers the advantage that both static conformation (from DEER distance measurements) and DNA flexibility (from the breadth of distance distributions) can be assayed accurately. On the other hand, since the AQ_2_ labeling scheme is dependent on both DNA strands coordinating copper, work using distance measurements to monitor DNA unwinding (e.g., during transcription) would be challenging.^119^

The advent of the rigid copper DNA label also offers several potential advantages. In EPR, this label can be combined with nitroxide or trityl labeling to improve sensitivity. For example, measurements utilizing the RIDME pulse sequence are sensitive to protein concentrations as low as 10 nM when rigid copper is paired with trityl labels.^121,122^ In these experiments, the fast longitudinal relaxation times and rigidity of the Cu­(II) label are crucial to enhance the modulation of the signal. Therefore, with copper-labeled DNA, it may be possible to monitor protein–DNA interactions at nanomolar concentrations. The label also has the potential to measure the relative orientations of the two spins by field dependent Q-band DEER measurements.^98^ Such information can potentially provide more information on processes such as DNA twisting.^31,35,38,123^ Finally, beyond EPR, this label can be a pathway for NMR paramagnetic relaxation enhancement and paramagnetic chemical shifts-based structural measurements^124–127^ and fluorescence quenching^128^ in the studies of protein–DNA interactions.

## Conclusions

In this work, we have made a significant improvement in the spin labeling methodology that can provide atomistic insight into DNA structural transitions and duplex flexibility.

The labeling strategy is nucleotide-independent and provides dramatic improvements in the resolution of DNA conformations and DNA flexibility by EPR. The new rigid Cu­(II) spin-label can be site-specifically incorporated into DNA oligonucleotides, and the positioning can be varied to examine site-specific changes in structure and flexibility. The label exploits Cu­(II) coordination to two 8-aminoquinoline moieties that are incorporated into complementary sites of the helix. The EPR reporters are therefore placed at the center of the helix, which maximize the sensitivity to DNA conformations. We show that Cu­(II) coordinates specifically to the AQ_2_ sites inside the DNA helix. Additionally, pulse EPR results indicate that Cu­(II) coordinates to four equatorial nitrogen atoms of the new motif and at least one oxygen from the solvent in the axial position.

Comparison with MD simulations suggests that the Cu­(II) label reports accurately on DNA distance constraints. Distance measurements from MD simulations are within 1.5 Å of the experimental Cu­(II)–Cu­(II) distance for each labeled duplex. Additionally, the new label provides distribution widths between 1 and 2 Å depending on the labeled sites, a significant improvement over other EPR probes. The precision and rigidity of the label provide a new technology to discern conformational and flexibility changes in DNA.

We use the new label to characterize the atomic level details of protein–DNA interactions when the DNA binds the restriction endonuclease *Eco*RV. We show that the label can accurately measure 2–3 Å level changes in DNA conformation and take advantage of this precision to provide confirming structural evidence in solution that *Eco*RV binding induces a DNA conformational change in both the presence and absence of metal ions. *Eco*RV binding also narrows the distance distribution, indicating a protein-induced constraint on DNA axial flexibility. In addition, we find that metal ions Ca­(II) and Lu­(III) induce a further narrowing of the distance distribution relative to that in the metal-free *Eco*RV–DNA complex, suggesting additional metal-induced reduction in DNA flexibility.

These catalytic centers (one in each *Eco*RV subunit) cannot form without distortion of the DNA imposed by the protein. The data in this paper show that (CuAQ)_2_–DNA faithfully recapitulates the required DNA bend and supports assembly of the metal-binding sites. Thus, our spin label is appropriate for studying the progress from the *Eco*RV–DNA encounter complex to the catalytic transition state, stopping short of DNA strand scission.

The bent DNA conformation in the protein–DNA complex differs markedly from the conformation of the free DNA, and the narrowing of the distance distributions as metals bind reflects the approach to the required precision of the catalytic transition state. Other experimental approaches provide little or no information about fluctuations in the DNA bend once the *Eco*RV–DNA complex has formed or about the distribution of bend angles (or point-to-point DNA distances) around their most probable (modal) values. The ability to measure these dynamic properties is a major strength of our approach.

## Supplementary Material



## Notes

Primary DEER data, CW-EPR and ESEEM of the Cu­(II) labeled DNA in the presence of protein, orientation selectivity analysis, details of the DNA breathing model, binding and catalytic inhibition by Lu­(III), and control DEER time trace fitting (PDF)
