# Supplementary material for: Caught in the Middle: A Rigid DNA Label That Provides an Incisive Picture of DNA Conformational Flexibility in Protein–DNA Complexes
Source: J Am Chem Soc. 2025 May 23;147(22):18723–36. doi: 10.1021/jacs.5c01823 (PMC12147125; doi:10.1021/jacs.5c01823)
Supplement: Supplementary file 1 [file ja5c01823_si_001.pdf]

SUPPORTING INFORMATION

Caught In the Middle: A Rigid DNA Label that Provides an Incisive Picture of DNA Conformational Flexibility in Protein-DNA Complexes

Joshua Casto[a‡], Shramana Palit[a‡], Anthony Little[a], Zikri Hasanbasri[a], Linda Jen-Jacobson[b], and Sunil Saxena[a]*

AUTHOR INFORMATION

[a]Department of Chemistry, University of Pittsburgh, Pittsburgh, Pennsylvania, 15260, United States
[b]Department of Biological Sciences, University of Pittsburgh, Pittsburgh, Pennsylvania 15260, United States.

Corresponding Author
[a*]Sunil Saxena - Department of Chemistry, University of Pittsburgh, Pittsburgh. PA 15260, USA. ORCID: 0000-0001-9098-6114, Phone (412) 624-8680. Email: sksaxena@pitt.edu.

Table S1. DEER collection parameters for data presented in the main text (Figures 4 and 6)

**Experiment**	n=9	n=11	n=13	n=17	n=9 +EcoRV	n=9 +EcoRV+Ca(II)	n=9 +EcoRV+Lu(III)
$(\frac{\pi}{2})\upsilon_A$ **[ns]**	12	12	12	12	12	12	12
$(\pi)\upsilon_A$ **[ns]**	24	24	24	24	24	24	24
$(\pi)\ \upsilon_B$ **[ns]**	20	20	20	20	20	20	20
**Att. [dB]**	4	4	4	4	4	4	4
**τ [ns]**	220	192	192	210	204	204	204
**T [ns]**	3160	3360	4320	5600	2598	2598	2598
Δt **[ns]**	20	24	24	32	18	18	18
**SRT [ms]**	1.5	1.5	1.5	1.5	1.5	1.5	1.5
**Points**	81	149	189	175	130	130	130
**SPP**	100	100	100	100	100	100	100
**Scans[a]**	2610	1590	2850	5112	1632	1704	2580
**λ [%]**	3.3	3.3	3.1	3.8	5.1	4.4	5.4
**SNR[b]**	77	56	52	28	58	52	52

a. 2-Step phase cycling was used for these data collections

b. SNR was calculated by dividing the modulation depth (λ) by the RMSD of the residual noise of the time trace as previously described.[1]

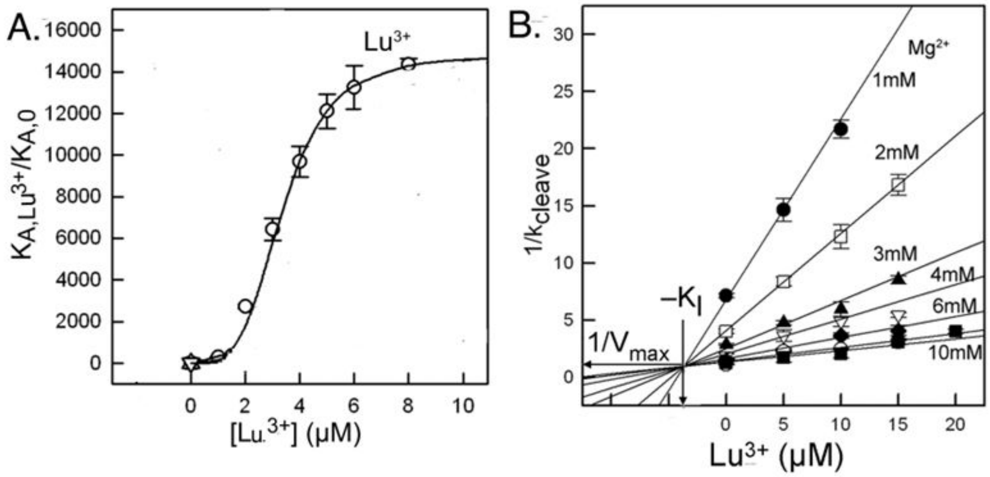

Figure S1. Lu(III) ions enhance EcoRV-DNA binding and competitively inhibit Mg(II)-catalyzed cleavage. A) Enhancement of EcoRV binding to GCAAAgatatcTTTCG in the absence of catalysis. This contains the same AAA flanking triplet used for the $(CuAQ)_2$-DNA experiments. We have shown that the sequence >3bp from the gatatc site has no effect on binding or cleavage. The sigmoidal dependence of binding enhancement [ratio of K_A in the presence of Lu(III) to $K_{A,0}$ in the absence of Lu(III)] shows that Lu(III) has a cooperative effect on stability of the EcoRV-DNA complex with half-maximum stimulation at 3.7 µM [Lu(III)]. The $K_{A,0}$ value was $3.9(\pm 0.2)x10^6$ M^{-1}. All values were determined in 20 mM potassium cacodylate, 0.22M KCl, 100 µg/ml bovine albumin, pH 7.4. B) Competitive inhibition of DNA cleavage by Lu(III). The DNA substrate was a 24-bp oligonucleotide 5'-CGCTGGAAAgatatcTTTGGAGGC-3' with the same flanking triplet as used for the binding studies in panel A. Reactions were carried out in the same binding buffer with the indicated concentrations of Mg(II). DNA cleavage was assayed by electrophoretic separation of the cleavage products, followed by autoradiography and densitometric quantitation of the products. The intersection of the curves (at $-K_I = -3.7$ µM, the concentration at half-maximum inhibition) indicates competitive inhibition.

ESEEM without D2O:

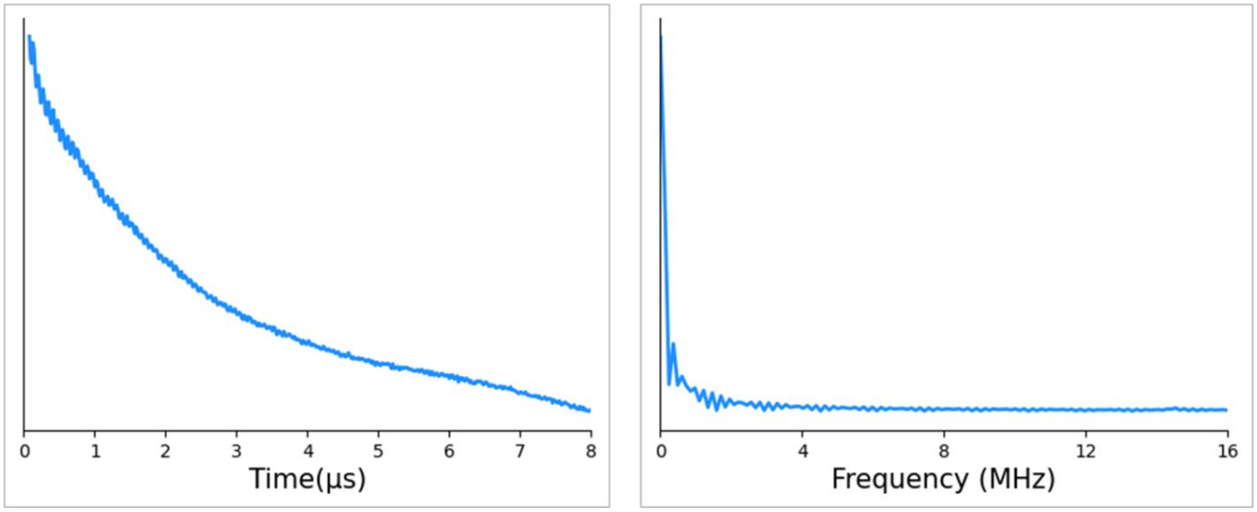

Figure S2. ESEEM data on the n=9 duplex in absence of D_2O. A) Time domain signal of the duplex. There are no modulations in the data indicating that there is no weakly coupled nitrogen to the Cu(II) center B) Frequency domain ESEEM spectrum of the duplex showing no peaks below 4 MHz suggesting Cu(II) is not weakly coupled to the nitrogen of neighboring bases.

Primary DEER Data:

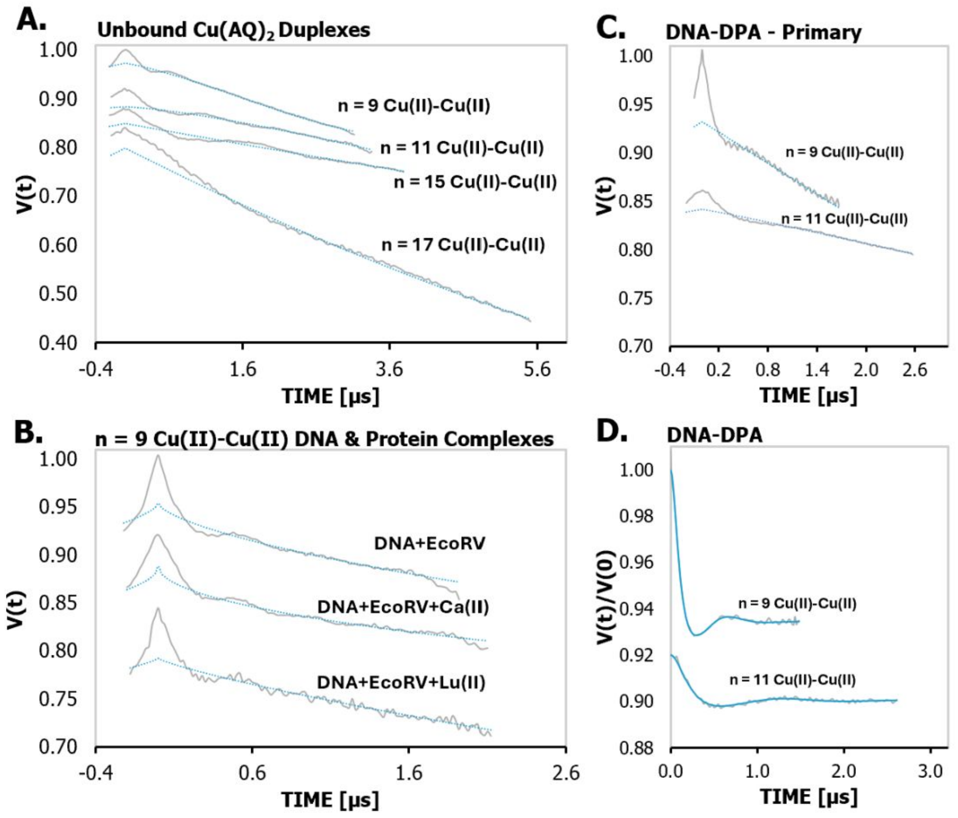

Figure S3. A) Raw DEER time traces for the data presented in the main text as shown for A) the Cu(II) labeled duplexes (cf. Figure 4) B) the n = 9 duplex with EcoRV and inhibitor samples (cf. Figure 6) and C) the reference DPA labeled sequences (cf. Figure 4). D) The background subtracted time traces for the DPA labeled reference duplexes. The dotted blue lines are the background fits obtained using DEERAnalysis.

Orientation Selectivity Analysis at X-Band:

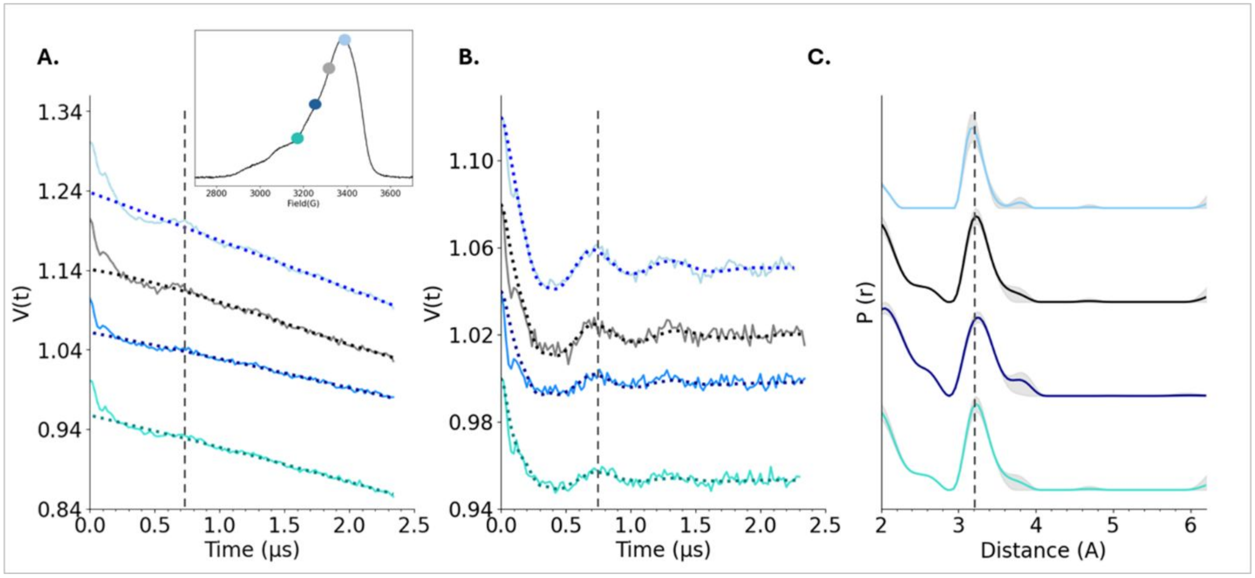

Figure S4. A) Primary DEER time traces obtained at different field positions for the Cu(AQ)$_2$ labeled n=9 duplex. The field swept electron spin echo Cu(II) absorption spectrum is shown in the inset. The colored circles on the spectra indicate DEER pump pulse positions. B) The background corrected time domain signal obtained at each field stacked for comparison. The dashed line highlights the matching modulations between the time traces. This result indicates that orientation selectivity is minimal for the Cu(II) label at X-Band. C) The distance distributions obtained at the different fields are largely similar.

To determine if there are orientation selectivity effects of the Cu(II) labeled DNA at X-Band, DEER was performed at four field positions indicated by vertical lines in the Cu(II) EPR spectrum of the n = 9 in the inset of Figure S4A. Figure S4B shows a comparison of the four background corrected time traces. Here we see that the modulation period of the time traces is in agreement, indicating there are no prominent orientation selectivity effects for this label at X-Band. In addition, the distance distributions are largely unchanged.

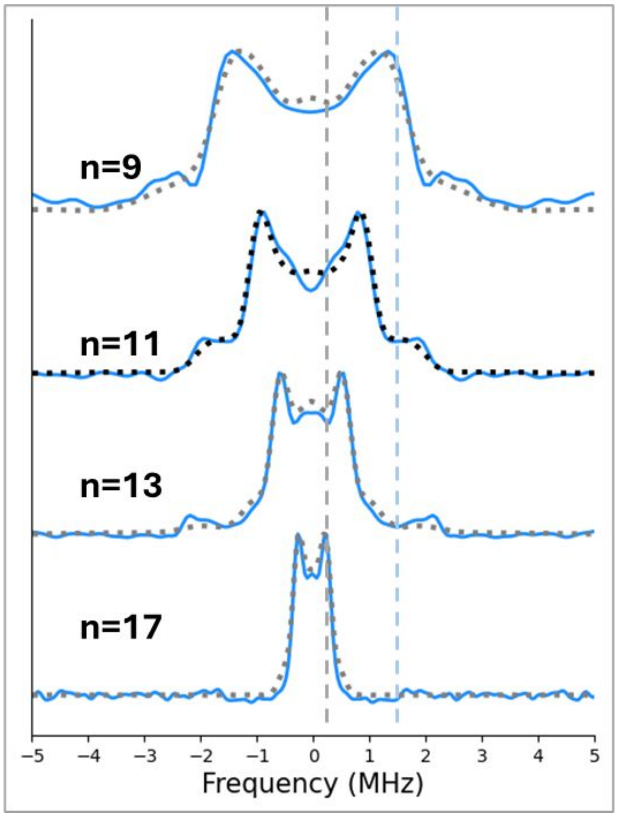

Figure S5. Pake pattern for the Cu(II) labeled duplexes as shown. The dotted spectra are the simulations from DeerAnalysis. The dashed lines highlight the differences in the peak position of the perpendicular orientation as the base pair separation increases from n = 9 to n =17.

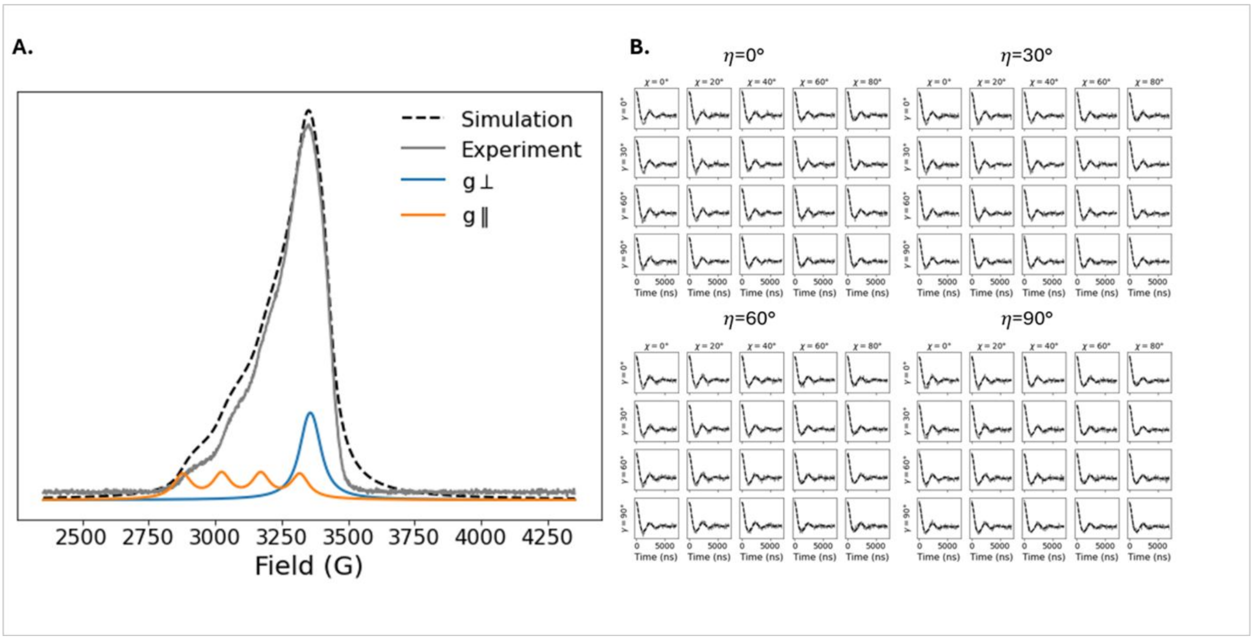

Figure S6. A) Experimental Echo Detected field swept spectrum of the Cu(II) label shown in grey overlaid with the analogous simulated spectrum (black dashed) computed by summation of the line shapes produced by randomly oriented spins. The line shape produced by spins oriented perpendicular and parallel are shown in blue and orange. There is sufficient overlap between the parallel and perpendicular regions at the field of maximum intensity of the field swept spectrum due to the lower g-anisotropy of this Cu(II) label. B) Simulated data which compares the DEER signals for complete excitation of all spins versus the signal that is anticipated by selective excitation using the experimental pulses.

In order to analyze sampling, we also performed in-silico simulations using procedures described recently [2,3]. Figure S6A shows the experimental and simulated FS-ESE spectrum. Overlaid on the figure are the signals from a spin with $g_{\perp}$ parallel to the magnetic field (blue) and another parallel to the magnetic field (orange). Due to the reduced g-anisotropy of this Cu(II) label, there is considerable overlap between the parallel and perpendicular regions near the maximum of the field swept spectrum. Therefore, DEER measurements performed at that position should adequately sample all orientations and thus, we expect little orientation selection. Figure S6B shows simulated data which compares the DEER signals for complete excitation of all spins versus the signal that is anticipated by selective excitation using experimental pulses. Details of the theory are provided elsewhere[2,3]. In the simulations the relative angles of the g-tensors are accounted for through three angles (χ, γ and η). Figure 4B shows data generated using 125 different combinations of these angles. In the simulation the standard deviations of these angles were 7°, 20° and 30° for χ, γ and η respectively. These were estimated from angular distributions observed for data on peptide acids that features a similar coordination[4]. Post simulation random noise was added to the time traces to lead to a SNR of 50:1. As is seen from Figure S6B overwhelmingly simulated traces are identical to full excitation.

Breathing Model:

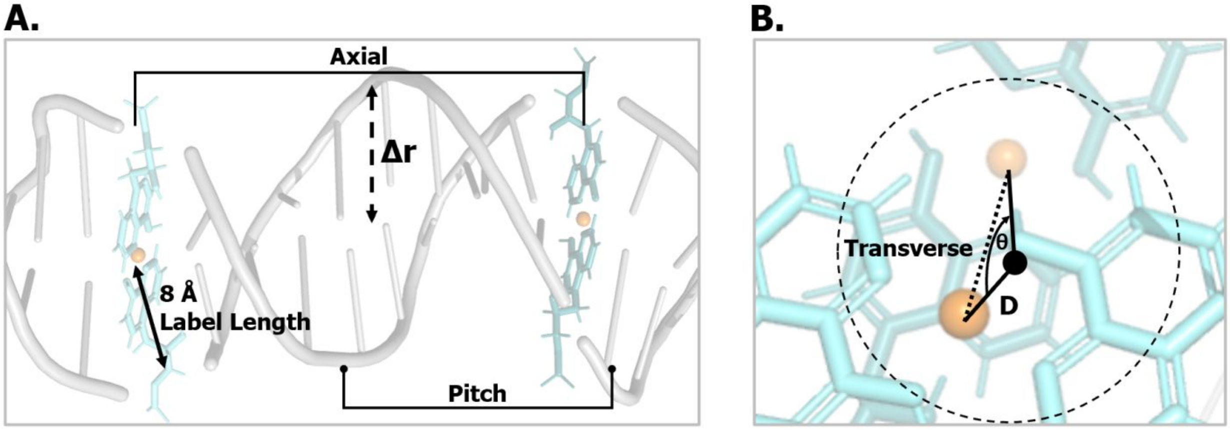

Figure S7. A modified breathing model of DNA was used to approximate the flexibility of the label. A) In the breathing model the radius of the helix, r, changes while keeping the length of the pitch constant. The axial length is determined by the distance between base pairs for B-DNA, 3.4 Å, and the base pair separation. Our model also incorporated the length of the linker created and optimized in PyMOL. The $Cu(AQ)_2$ shown is placed inside the helix within a n = 9 base pair separation B) The offset between the Cu(II) centers, otherwise the transverse length, is also used in the model to approximate the distance between the Cu(II) labels. Here D is deviation of the Cu(II) from the center of the helix assuming the radius is 5.6 Å. The calculation of D used the estimated length of linker from the $Cu(AQ)_2$ structure created in PyMOL. The angle θ is the relative angle of the Cu(II) to each other. The angle is dependent on the base pair separation between Cu(II) since the helix does a full turn every 10.4 base pairs.

To estimate the flexibility of the Cu(II) label in the duplexes a previously reported breathing model of DNA was used.[5] The DNA breathing model assumes the duplex is a cylindrical rod with a radius that fluctuates. The subtle changes in the radius throughout the duplex engenders small fluctuations in the distance between base pairs, while the pitch of the helix remains constant (cf. Figure S7A). The model includes adjustable parameters for the distance between Cu(II) labels and the flexibility of the radius, Δr. The flexibility of the radius was adjusted to fit the distance distribution from the model to those measured by the experiments. The Δr used to best fit the model to each experimental distance provides insight on the flexibility of the label.

In the model we incorporated the length of the label into the distance between Cu(II) sites.[5–7] The length of the label is relevant to the transverse length between the Cu(II) sites. Figure S7B depicts the transverse length between the Cu(II). The transverse length is determined by radius offset of each Cu(II) from the center of the helix, D, and the relative angle, θ, of the two spins using the center of the helix as the angle origin. To determine the transverse length we created a structure of the $Cu(AQ)_2$ label in Pymol.[8] The four Cu(II)-N bonds were set as 2 Å.[4,9,10] The remaining bonds lengths and angles in the complex were optimized by Pymol using an automated function.[8] Next, we took the equilibrated structure of the native sequence and removed the nucleotides at the target labeled sites. The native duplex was equilibrated with molecular dynamics

(MD). Details of the MD simulations are provided later in the methods section. The $Cu(AQ)_2$ structure was placed at these sites with the phosphoramidite aligned with the duplex backbone. The length of the label was measured to be ca. 8 Å from the Cu(II) center to the C' carbon of the phosphonamidite linker.

The radius offset, D, was obtained by subtracting the length of the helix by the radius of the helix. This was calculated assuming the radius of the helix is 5.6 Å. Next, the angle θ was obtained using the following equation:

$$\theta = \theta_0 + \frac{2\pi n}{N} \quad \textit{(Equation 5-1)}$$

Here, θ_0 is the initial angle at zero base pair separation, e.g. 180°, n is the base pair separation, and N is the number of base pairs in average helical turn, 10.4. The transverse length was then calculated as:

$$transverse = 2D\sin\frac{\theta}{2} \quad \textit{(Equation 5-2)}$$

The axial length was determined by multiplying the respective base pair separation by the average distance between base pairs in a B-DNA, 3.4 Å.[11] Finally, the transverse and axial lengths were combined to determine the distance between Cu(II) sites to use in the model.

$$Length = \sqrt{transverse^2 + axial^2} \quad \textit{(Equation 5-3)}$$

More explicit equations and calculations for the length measurements and the breathing model are provided in earlier papers.[5–7] These steps were carried out for each base pair separation of the labeled duplexes discussed in the main text. The standard deviation of the radius in the breathing model was then varied until the modeled distance distributions fit the experimental distributions.

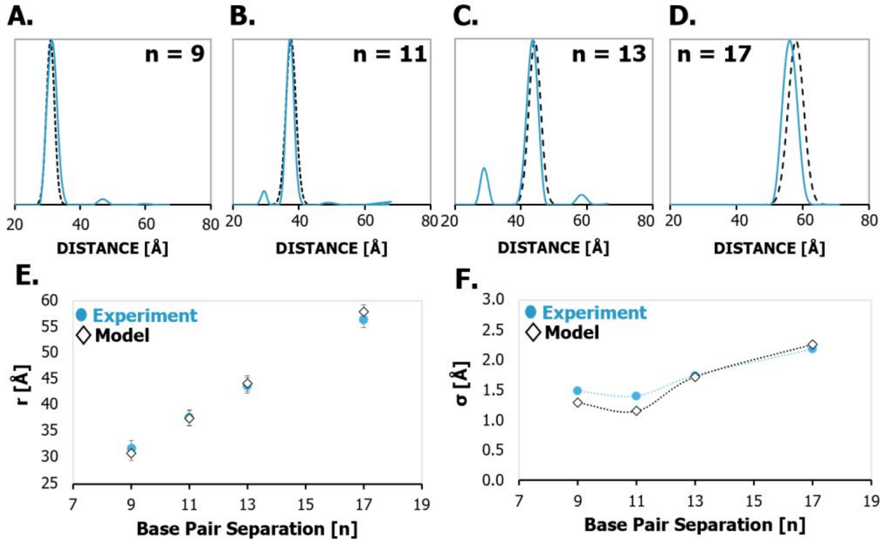

Figure S8. A-D) Comparison of the modified DNA breathing model distance distributions (black) to the Cu(II)-Cu(II) distances (blue) for each respective base pair separation in the duplex, n. The model distributions were obtained using a Δr of 0.46 Å for the helical radius. Native B-DNA has

a typical Δr of 0.65 Å. E) Comparison of the most probable distances form the model and experiment plotted as a function of base pair separations. The distances are within 1.5 Å across all duplexes. F) Comparison σ from the model and experiment plotted as function of base pair separation. The trend in the change of distribution width as the base pair separation increases agree well between experiment and model.

We estimated the flexibility of the label by utilizing the breathing model,[5,12] which has previously been shown to capture the dynamics of DNA of similar lengths. Figures S8A-D show the comparison between the experimental distance distributions and those created using the breathing model. Here the model distributions agree well with the experimental distributions. Figure S8E highlights the reasonable agreement between the most probable distance of the model and experiment for each duplex. Figure S8F shows the similarity in change of flexibility across base pair separations between the experiment and model.

In our breathing model a label flexibility of 0.45 Å was used to fit the model distributions to the experimental. The same model estimated a flexibility of 1.65 Å for the DPA label and 0.1 for the **ç** label. Indeed, the model is consistent with the narrower distribution obtained using the Cu(II) label. Such rigidity is advantageous to deconvolute crucial biophysical information associated with changes in distributions widths from the contributions to the width from the label itself.

Cumulatively the results from breathing model combined with MD results exemplify the ability of the Cu(II) label to report straightforwardly on relevant duplex distance constraints and changes in duplex flexibility and resolve bimodal distributions. The results also illustrate the physical reasoning behind the remarkable narrow distribution width compared to the DPA and nitroxide labels (cf. Figure 4B).

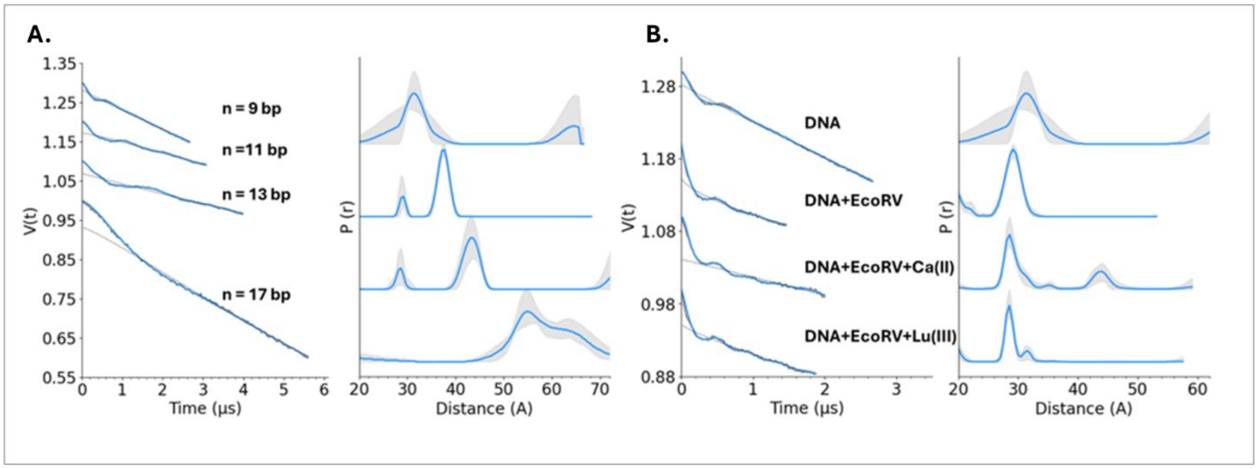

Figure S9. The distance distributions and fits obtained via ComparativeDeerAnalyzer (CDA)[13] for each of the DNA samples using Consensus. . The grey shaded regions are the validated distance distributions. A) Distance distributions and fits for each of the duplexes. The distance distributions match reasonably well with the distributions obtained via DeerAnalysis. The distribution for the n=17 bp duplex is slightly different probably due to difficult background fitting. B) Distance distributions and fits for n=9 duplex compared to DNA with protein and DNA with protein in presence of metal ions. The most probable distance decreases by 3 Å when the DNA is in the presence of EcoRV and EcoRV coordinated to Ca(II) or Lu(III) which is consistent with the distributions obtained with Tikhonov regularization via DEERAnalysis

Table S2. Most probable distances calculated by modulation period, DeerAnalysis and Consensus.

	From Modulation Period (Å)	*DeerAnalysis (Å)*	*Consensus (Å)*
n = 9	33	32	32
n = 11	38	37	37
n = 13	45	44	44
n = 17	58	57	56
DNA+EcoRV	30	30	30
DNA+EcoRV+Ca(II)	30	29	29
DNA+EcoRV+Lu(III)	30	29	29

Cu(II) Coordination to EcoRV:

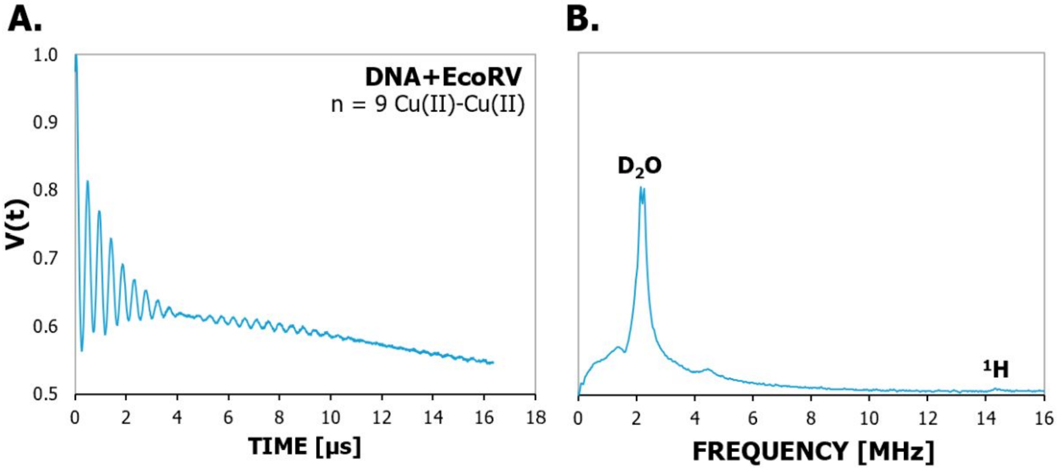

Figure S10. ESEEM data of the Cu(II) labeled duplex in the presence of EcoRV. The A) time domain signal and B) frequency domain spectrum is identical to the ESEEM data of the unbound Cu(II) labeled DNA in the main text (cf. Figure 3). Here the doublet peak ca. 2.4 MHz with a broad shoulder spanning from 0-4 MHz is characteristics of axially coordinated D_2O to Cu(II), as explained in the main text.

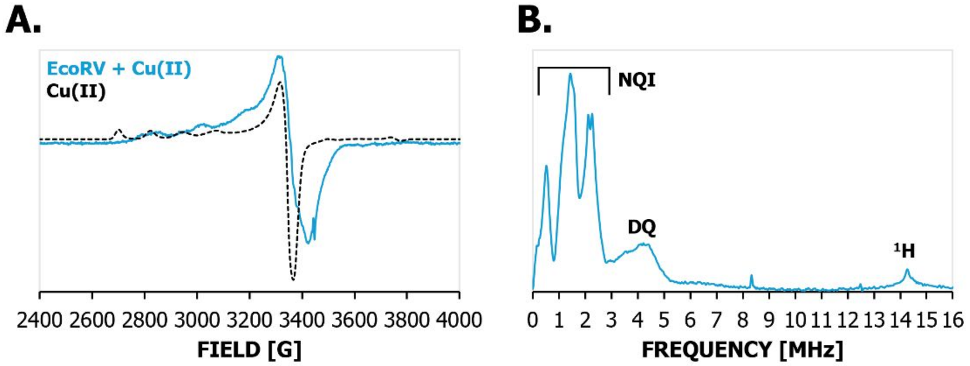

Figure S11. A) The CW spectra of free Cu(II) (black dashed) and Cu(II) in the presence of EcoRV (blue solid). There are clear differences between the spectra. Therefore, Cu(II) must be coordinating to EcoRV. B) The ESEEM frequency domain spectra of Cu(II) in the presence of EcoRV. The spectrum has three peaks present below ca. 2 MHz and a broad peak at 4 MHz. These features are characteristic of the nuclear quadrupole interaction (NQI) and double quantum (DQ) associated with Cu(II) coordinated to histidine resiudes.[14–16] Wildtype EcoRV has five native histidine residues. Samples were prepared with one equivalent of Cu(II) per EcoRV dimer.

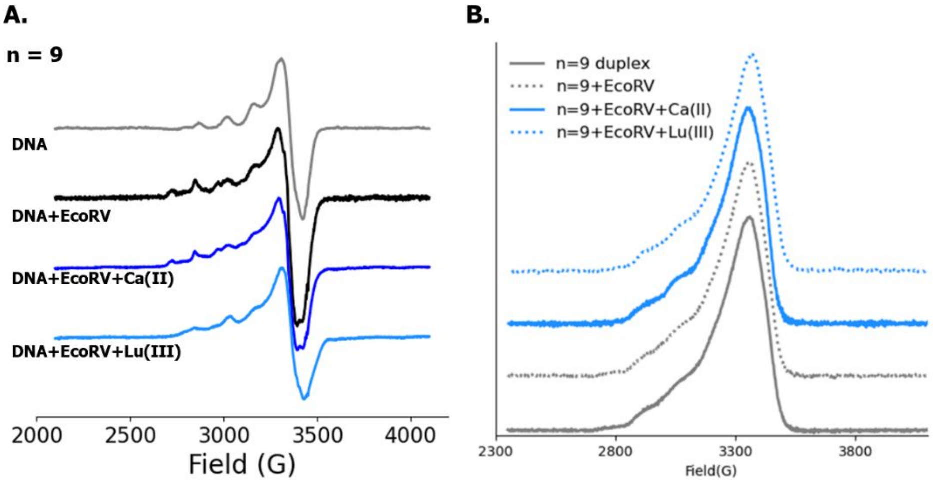

Figure S12. A) The CW-EPR spectra and B) The field swept electron spin echo spectrum for n=9 duplex and the n=9 duplex in presence of EcoRV with and without the metal ions Ca(II) and Lu(III). The spectra and field sweep show various Cu(II) components in the presence of EcoRV and the duplex. These data suggest there is a nonspecific binding of Cu(II) to EcoRV amino acids, such as its native histidine residues.

Tikhonov Regularization impact on Standard Deviation Analysis:

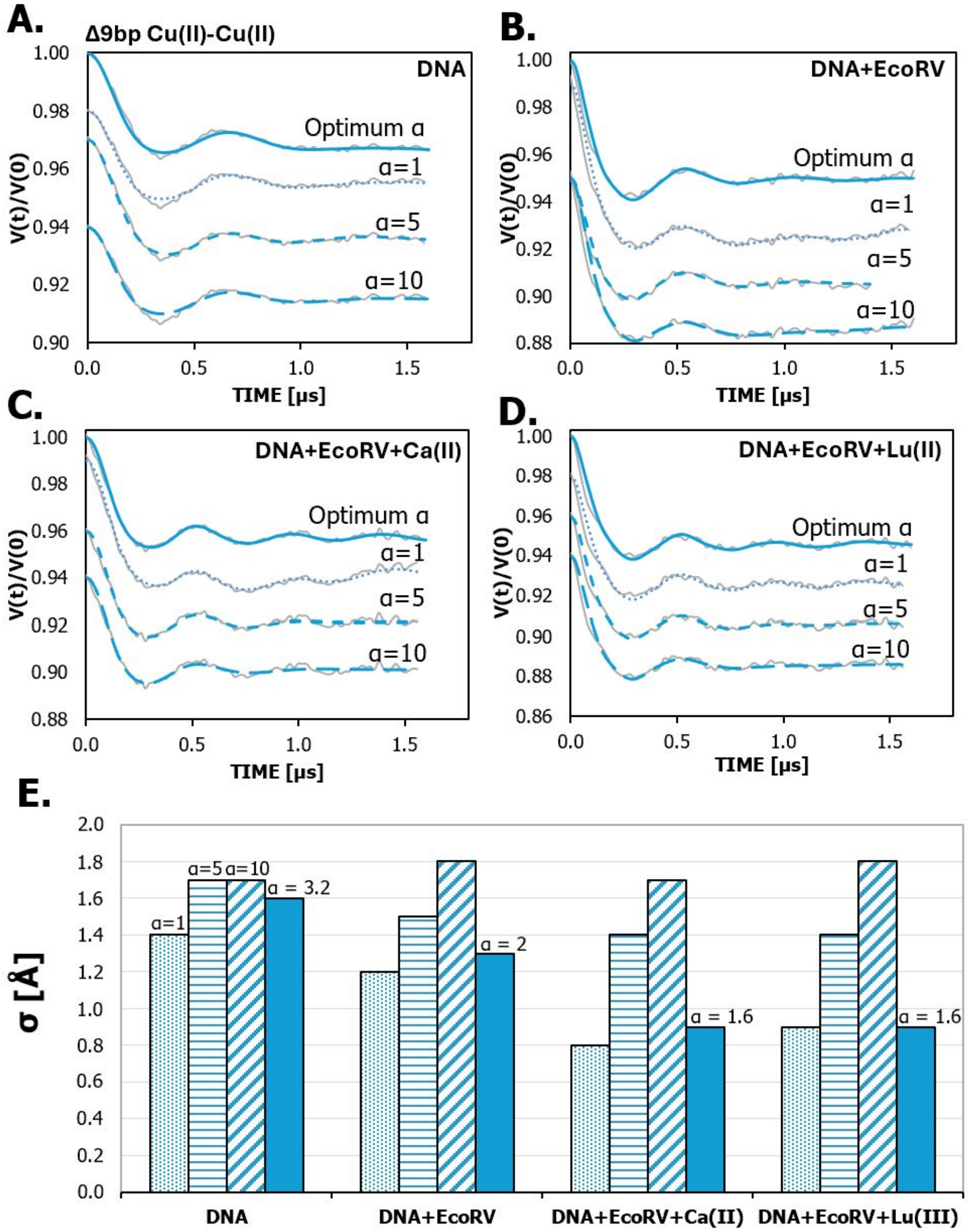

Figure S13. Background subtracted DEER time traces for various samples using the Δ9bp Cu(II) labeled duplex as described in each figure. Each time trace was obtained with Tikhonov regularization in DEERAnalysis by using variable regularization parameter, α, as shown (1, 5, 10, and the optimum value from the L-curve for each time-trace). A) The Cu(II) labeled duplex B) the duplex in the presence of EcoRV C) the duplex in the presence of EcoRV and Ca(II) and D) the duplex in the presence of EcoRV and Lu(III). For each data set the smaller the α value the better the fit due to the rigidity of the label resulting in clear modulations in the time-trace. E) A comparison of the standard deviations of the distributions from each time trace in A-D with the

various α-values as shown. The α-values of 1 agrees well standard deviations obtained using the optimal α-value.

When using Tikhonov regularization to fit DEER time-traces, the resulting distribution width is dependent on the regularization parameter, α, used. The regularization parameter optimizes the smoothness of the fit to ensure the noise in the time trace is not being considered in the fit. As described in the main text, the standard deviations for the Cu(II) labeled duplex distance distributions get smaller as the duplex binds to the protein, and narrow furthers when a metal inhibitor is introduced. Therefore, to make sure the standard deviation trends in our data presented in the main text were not coincidental with the chosen α values we varied α around the optimal α for each time trace from the L-curve.

Figure S13 shows the time trace fits for the n=9 Cu(II) labeled duplexes respectively as the a-value is varied from 1 to 10. The data presented in the main text used an optimal α-value chosen from the L-Curve. The sample composition for each data set is described in each figure. Figures S13 A-D qualitatively show the fits using different α-values agree well with each other. Figure S13D compares the standard deviations obtained using the different α-values. In Figures S13D the standard deviations follow the same trend and are in reasonable agreement with each other up to a α=10. The standard deviations deviate from the trend slightly at α=10 because the fit is becoming oversmoothed and not capturing the narrow modulations efficiently.

Table S3: Comparison of most probable distances reported by rigid DNA labels in literature for similar base pair separations. $Cu(AQ)_2$ and Cu(II)-DPA[6] are placed within the helix. **Ç**[5] is placed on the same face of the DNA, ^{ExIm}U[17] is on the opposite face of the DNA strand for n=8 but on the same face for n=11. **Ǵ** is placed on opposite face for n=9. [18]

	B-DNA (Å) (MD)	*$Cu(AQ)_2$ (Å)*	*Cu(II)-DPA (Å)*	***Ç*** *(Å)*	***Ǵ*** *(Å)*	*^{ExIm}U (Å)*
n=8	27		27	29		40
n=9	30	32	31	30	37	
n=11	36	37	39	35		36
n=13	44	44		42		

References:

(1) Abdullin, D.; Brehm, P.; Fleck, N.; Spicher, S.; Grimme, S.; Schiemann, O. Pulsed EPR Dipolar Spectroscopy on Spin Pairs with One Highly Anisotropic Spin Center: The Low-Spin FeIII Case. *Chem. - Eur. J.* **2019**, *25* (63), 14388–14398. https://doi.org/10.1002/chem.201902908.

(2) Hasanbasri, Z.; Moriglioni, N. A.; Saxena, S. Efficient Sampling of Molecular Orientations for Cu(II)-Based DEER on Protein Labels. *Phys. Chem. Chem. Phys.* **2023**, *25* (19), 13275–13288. https://doi.org/10.1039/D3CP00404J.

(3) Bogetti, X.; Hasanbasri, Z.; Hunter, H. R.; Saxena, S. An Optimal Acquisition Scheme for Q-Band EPR Distance Measurements Using Cu $^{2+}$ -Based Protein Labels. *Phys. Chem. Chem. Phys.* **2022**, *24* (24), 14727–14739. https://doi.org/10.1039/D2CP01032A.

(4) Gamble Jarvi, A.; Sargun, A.; Bogetti, X.; Wang, J.; Achim, C.; Saxena, S. Development of Cu2+-Based Distance Methods and Force Field Parameters for the Determination of PNA Conformations and Dynamics by EPR and MD Simulations. *J. Phys. Chem. B* **2020**, *124* (35), 7544–7556. https://doi.org/10.1021/acs.jpcb.0c05509.

(5) Schiemann, O.; Sigurdsson, S. Th.; Cekan, P.; Denysenkov, V.; Marko, A.; Prisner, T. F.; Margraf, D. Conformational Flexibility of DNA. *J. Am. Chem. Soc.* **2011**, *133* (34), 13375–13379. https://doi.org/10.1021/ja201244u.

(6) Ghosh, S.; Lawless, M. J.; Brubaker, H. J.; Singewald, K.; Kurpiewski, M. R.; Jen-Jacobson, L.; Saxena, S. Cu2+-Based Distance Measurements by Pulsed EPR Provide Distance Constraints for DNA Backbone Conformations in Solution. *Nucleic Acids Res.* **2020**, 1–11. https://doi.org/10.1093/nar/gkaa133.

(7) Mathew-Fenn, R. S.; Das, R.; Harbury, P. A. B. Remeasuring the Double Helix. *Science* **2008**, *322* (5900), 446–449. https://doi.org/10.1126/science.1158881.

(8) Delano, W. L. PyMOL: An Open-Source Molecular Graphics Tool.

(9) Ghosh, S.; Casto, J.; Bogetti, X.; Arora, C.; Wang, J.; Saxena, S. Orientation and Dynamics of Cu2+based DNA Labels from Force Field Parameterized MD Elucidates the Relationship between EPR Distance Constraints and DNA Backbone Distances. *Phys. Chem. Chem. Phys.* **2020**, *22* (46), 26707–26719. https://doi.org/10.1039/d0cp05016d.

(10) Bogetti, X.; Ghosh, S.; Gamble Jarvi, A.; Wang, J.; Saxena, S. Molecular Dynamics Simulations Based on Newly Developed Force Field Parameters for Cu2+ Spin Labels Provide Insights into Double-Histidine-Based Double Electron-Electron Resonance. *J. Phys. Chem. B* **2020**, *124* (14), 2788–2797. https://doi.org/10.1021/acs.jpcb.0c00739.

(11) Olson, W. K.; Gorin, A. A.; Lu, X. J.; Hock, L. M.; Zhurkin, V. B. DNA Sequence-Dependent Deformability Deduced from Protein-DNA Crystal Complexes. *Proc. Natl. Acad. Sci. U. S. A.* **1998**, *95* (19), 11163–11168. https://doi.org/10.1073/pnas.95.19.11163.

(12) Marko, J. F. Stretching Must Twist DNA. *Europhys. Lett.* **1997**, *38* (3), 183–188. https://doi.org/10.1209/epl/i1997-00223-5.

(13) Worswick, S. G.; Spencer, J. A.; Jeschke, G.; Kuprov, I. Deep Neural Network Processing of DEER Data. *Sci. Adv.* **2018**, *4* (8). https://doi.org/10.1126/SCIADV.AAT5218.

(14) Silva, K. I.; Michael, B. C.; Geib, S. J.; Saxena, S. ESEEM Analysis of Multi-Histidine Cu(II)-Coordination in Model Complexes, Peptides, and Amyloid-β. *J. Phys. Chem. B* **2014**, *118* (30), 8935–8944. https://doi.org/10.1021/jp500767n.

(15) Casto, J.; Palit, S.; Saxena, S. *PELDOR to the Metal: Cu(II)-Based Labels Put a New Spin on Distance Measurements*; Springer Vienna, 2024; Vol. 55. https://doi.org/10.1007/s00723-024-01658-8.

(16) Quintanar, L.; Millhauser, G. L. *EPR of Copper Centers in the Prion Protein*, 1st ed.; Elsevier Inc., 2022; Vol. 666. https://doi.org/10.1016/bs.mie.2022.02.003.

(17) Gophane, D. B.; Endeward, B.; Prisner, T. F.; Sigurdsson, S. Th. Conformationally Restricted Isoindoline-Derived Spin Labels in Duplex DNA: Distances and Rotational Flexibility by Pulsed Electron–Electron Double Resonance Spectroscopy. *Chem. – Eur. J.* **2014**, *20* (48), 15913–15919. https://doi.org/10.1002/chem.201403726.

(18) Heinz, M.; Erlenbach, N.; Stelzl, L. S.; Thierolf, G.; Kamble, N. R.; Sigurdsson, S. T.; Prisner, T. F.; Hummer, G. High-Resolution EPR Distance Measurements on RNA and DNA with the Non-Covalent Ǵ Spin Label. *Nucleic Acids Res.* **2020**, *48* (2), 924–933. https://doi.org/10.1093/nar/gkz1096.
